# pH-responsive and dual-dynamically crosslinked metal-phenolic hydrogel for synergistic macrophage and Th17/Treg reprogramming in diabetic wounds

**DOI:** 10.1186/s12951-025-03987-7

**Published:** 2026-01-12

**Authors:** Yuheng Liao, Yanzhi Zhao, Chenyan Yu, Zhenhe Zhang, Yonggang Yuan, Lizhi Ouyang, Weixian Hu, Shengming Zhang, Fawwaz Al-Smadi, Bobin Mi, Mengfei Liu, Hui Li, Guohui Liu

**Affiliations:** Department of Orthopedics, Union Hospital, Tongji Medical College, Huazhong University of Science and Technology, No. 1277 Jiefang Avenue, Hubei, 430022 Jianghan District, Wuhan China

**Keywords:** Immune regulation, Metal phenolic network, Adaptive immunity, Specific immunity, Wound healing

## Abstract

In recent years, there has been a progressive increase in the cumulative number of individuals afflicted with diabetic wounds, elevating this condition to a global public health concern of significant magnitude. The disruption of the immune microenvironment is recognized as a critical factor impeding the healing process of diabetic wounds. Nevertheless, existing research has predominantly concentrated on innate immune regulation, particularly targeting macrophages, while often overlooking adaptive immunity, which plays an equally vital role within the immune microenvironment. To address both innate and adaptive immune remodeling in diabetic wounds, the CSp-OxD@Cu^2+^/G hydrogel system has been developed. This system features a dual-dynamically crosslinked structure formed through Schiff base and borate ester bonds, with Cu^2+^/G nanoparticles generated via the chelation of guaiacol with copper ions. Upon application to diabetic wounds, the hydrogel system’s self-healing and adhesive properties provide an effective physical barrier. Concurrently, the borate ester bonds gradually respond to H^+^ in the local environment, dissociating and facilitating the slow release of Cu^2+^/G nanoparticles, thereby reprogramming macrophages and Th17/Treg cells to concurrently regulate both innate and adaptive immune processes. This therapeutic system, characterized by its comprehensive immune regulatory effects, offers a novel platform for the management of diabetic wounds.

**Graphical Abstract:**

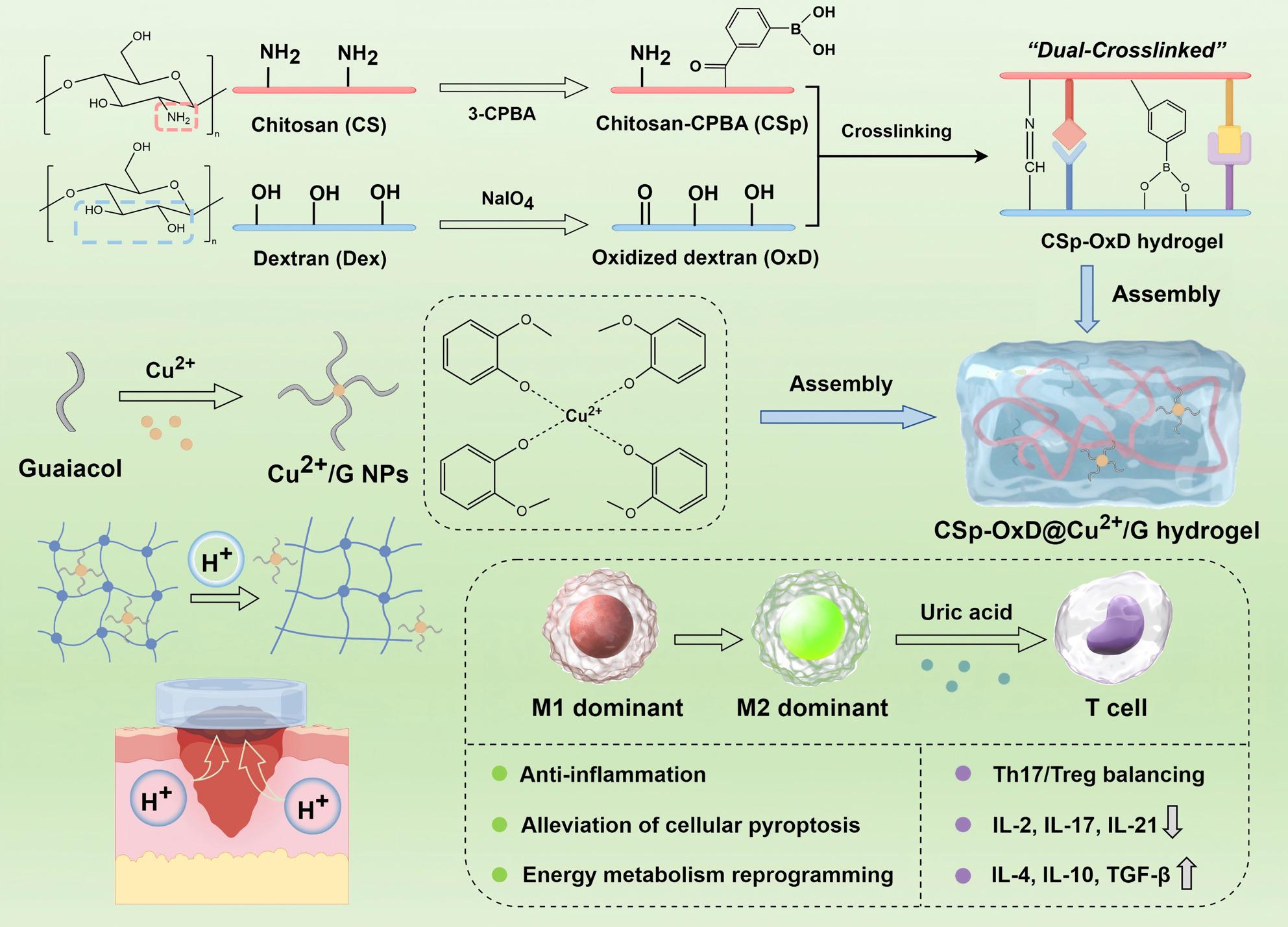

**Supplementary Information:**

The online version contains supplementary material available at 10.1186/s12951-025-03987-7.

## Introduction

Diabetic wounds represent one of the most prevalent and severe complications in patients with diabetes, marked by persistent inflammation, impaired angiogenesis, aberrant extracellular matrix remodeling, and a propensity for recurrent infections [1, 2]. These conditions frequently result in chronic non-healing ulcers, which may escalate to severe outcomes such as amputation [3, 4]. While current treatment modalities, including debridement, glycemic control, and bio-dressings, have led to partial improvements in clinical outcomes, the molecular mechanisms underlying diabetic wound healing remain inadequately understood [5, 6]. Notably, dysfunction within the immune microenvironment is recognized as a critical factor impeding the repair process. Contemporary research in this domain predominantly investigates the regulatory function of macrophage polarization, specifically the conversion between M1 and M2 phenotypes, within the inflammatory milieu of diabetic wounds [7–9]. It is crucial to acknowledge that diabetic wounds also exhibit dysfunction in T cell differentiation, marked by an elevated Th1/Th17 ratio and a diminished Treg ratio [10–12]. The interplay between T cells and macrophages may exacerbate the persistent cycle of ‘chronic inflammation-repair imbalance’ [13, 14]. A treatment paradigm with comprehensive immune regulatory capabilities presents a novel platform for the management of diabetic wounds.

Guaiacol, also known as 2-methoxyphenol, has been extensively utilized in the pharmaceutical industry since the mid-20th century, serving as an expectorant and cough suppressant, as well as a precursor in the synthesis of anti-inflammatory and antibacterial agents such as potassium guaiacol sulfonate and ibuprofen guaiacol ester [15–17]. Our research has identified that guaiacol not only exhibits anti-inflammatory properties in diabetic wounds but also plays a role in modulating the immune microenvironment. Furthermore, we have synthesized Cu^2+^/guaiacol aldehyde nanoparticles by chelating guaiacol with metal ions, creating a multifunctional therapeutic platform that integrates sustained-release capabilities, antibacterial synergy, and immune modulation. The formation of Cu^2+^/guaiacol nanoparticles significantly enhances the chemical stability of guaiacol and extends the duration of its local therapeutic effect at the wound site. Cu^2+^ itself is endowed with broad-spectrum antibacterial activity and possesses certain immunomodulatory properties, which contribute to reducing the potential biotoxicity of guaiacol and improving its biocompatibility.

Due to their notable stability, high drug encapsulation efficiency, favorable biocompatibility, and ease of application for localized wound management, various multifunctional hydrogel dressings have been developed for the treatment of diabetic wounds [18–21]. These hydrogel designs are ingenious and practical, demonstrating the multifunctionality of biomaterials across various dimensions, such as photothermal, electrochemical, microneedle structures, and gas therapy [3]. They also typically exhibit various bioactivities, including promoting proliferation, reducing programmed cell death (e.g. apoptosis, pyroptosis, ferroptosis), immune regulation, anti-scarring, reactive oxygen species scavenging, and microenvironment detection [22, 23]. However, many of these dressings are passive and do not adequately adapt to the dynamic nature of the wound healing process. Consequently, it is imperative to develop smart materials capable of responding to the wound microenvironment at different stages of healing [22, 24–26]. Borate ester bonds formed between diols and boric acid present several advantages, including facile modification techniques, mild reaction conditions, and excellent adhesive properties [27, 28]. Furthermore, these bonds demonstrate rapid rates of bond dissociation and reformation, which can be influenced by low pH levels and glucose, thereby making them responsive to the microenvironment characteristic of diabetic ulcers [29–31]. This structure has been incorporated into hydrogels by numerous studies, enabling the construction of pH-responsive smart platforms tailored to the requirements of diabetic wound healing [32–34]. Nevertheless, hydrogels composed solely of borate ester bonds exhibit rapid degradation in the mildly acidic conditions typical of diabetic wounds, necessitating frequent dressing changes, which adversely affects drug delivery and wound management [35, 36]. To address this issue, we synthesized CSp-OxD hydrogels by grafting 3-carboxyphenylboronic acid onto chitosan (CSp) and subsequently reacting it with oxidized dextran (OxD). During this synthesis, the amino groups (-NH_2_) of chitosan form Schiff bases with the carbonyl groups of oxidized dextran, while the boronic acid groups interact with the hydroxyl groups in the unoxidized dextran to form borate ester bonds. This dual interaction imparts pH responsiveness to the hydrogel and stabilizes its structure in conjunction with the Schiff bases.

In this study, we incorporated Cu^2+^/G nanoparticles into a CSp-OxD hydrogel to develop the CSp-OxD@Cu^2+^/G hydrogel therapeutic system (Scheme 1, Figure S19). Upon application to diabetic wounds, the slightly acidic environment facilitates the degradation of the CSp-OxD@Cu^2+^/G hydrogel, leading to the release of Cu^2+^/G NPs, which subsequently liberate guaiacol and copper ions. Our research will concentrate on the immune microenvironment modulation capabilities of this therapeutic system, with a specific focus on macrophages and Th17/Treg cells. We will employ metabolomics analysis to explore the interactions between these two immune cell types. In conclusion, the CSp-OxD@Cu^2+^/G hydrogel demonstrates exceptional immune regulatory properties, effectively remodeling both macrophage and Th17/Treg cells. Additionally, it exhibits pH responsiveness and antimicrobial activity, rendering it a promising therapeutic strategy for diabetic wounds. This study not only offers novel insights into the immunological mechanisms underlying diabetic wound healing but also establishes a theoretical foundation for the development of innovative therapeutic strategies targeting the immune microenvironment.

**Fig. 1:**
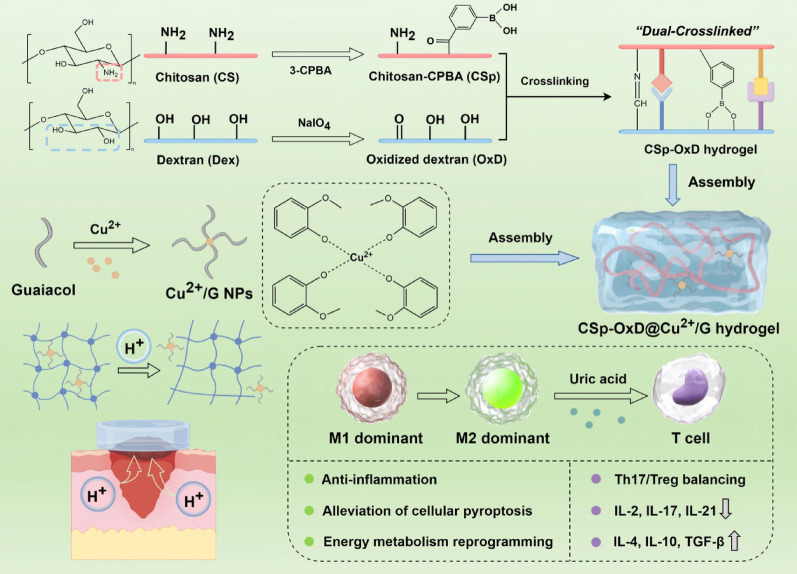
Schematic diagram of CSp-OxD@Cu^2+^/G hydrogel application in diabetic wound treatment

## Experimental methods

### Preparation of Cu^2+^/G NPs

0.135 g CuCl_2_ and 0.124 g guaiacol were dissolved in 30 mL distilled water, stirred for at room temperature for 2 h, washed twice with distilled water (12,000 rpm, 5 min), freeze-dried for 24 h, and the black particles obtained were collected.

### Characterization of Cu^2+^/G NPs

Using a Transmission Electron Microscope (TEM, Talos F200S, Thermo Fisher Scientific, USA), the microstructural morphology and elemental mapping of Cu^2+^/G nanoparticles were investigated. Using X-ray photoelectron spectroscopy (XPS, Thermo Kalpha, USA), the crystal structure of Cu^2+^/G nanoparticles was analyzed. The particle size distribution and Zeta potential of were examined using Malvern Zetasizer Nano ZS90 (Malvern Panalytical, UK).

### Preparation of CSp-OxD@Cu^2+^/G hydrogel

*Preparation of 3-carboxyphenylboronic acid grafted chitosan (CSp).* A homogeneous solution was formed by dissolving 1 g of chitosan (CS) in 100 mL of 0.3% acetic acid with stirring. CPBA, EDC-HCl, and NHS were dissolved entirely in 15 mL of ethanol according to a 1:2:2.5:2.5 molar ratio. They were then added to the above CS solution. The mixture was stirred for 24 h and dialysed in distilled water for 3 days followed by freeze-drying for 3 days.

#### Preparation of oxidized dextran (OxD)

To make the solution, dissolve 10 g of dextran in 100 ml of distilled water. A 100 g/L sodium periodate solution was prepared, with a dextran to NaIO_4_ molar ratio of 2:1. The mixture was then combined with dextran and allowed to react in the dark for 6 h at room temperature. To stop the reaction, 2 mL of ethylene glycol was added. Dialysis was conducted on the product using 3500 Da MwCO bags, with daily dialysate changed until it became clear. The solution was then frozen at −80 ℃ overnight and freeze-dried to yield a white powder.

#### Preparation of CSp-OxD@Cu^2+^/G hydrogel

For the preparation of CSp-OxD, hydrogels were formulated by mixing 3% (w/v) solutions of OxD and CSp. For the preparation of CSp-OxD@Cu^2+^/G, the Cu^2+^/G NPs were mixed well with OxD solution before adding CSp solution.

### Characterization of CSp-OxD@Cu^2+^/G hydrogel

*Microstructure and molecular structure characterization*. Using SEM with a Sigma 300 (Carl Zeiss AG, Germany), the microstructure of CSp-OxD hydrogel and CSp-OxD@Cu^2+^/G hydrogel was tested. The molecular structure of CSp was tested with H1NMR using a Bruker Avance NEO 600 (Bruker, Germany). Using a Nicolet iS20 from Thermo Fisher Scientific, USA, the molecular structure of the CSp-OxD hydrogel was tested via FTIR.

*Rheological test*. The hydrogels were assessed using a Mars40 rheometer (Haake Technik GmbH, Germany) under parallel plate conditions. Time scans were conducted at a strain of 1% and a frequency of 1 Hz. Using a CMT6103 universal testing machine (MTS Systems Corporation, China), compression tests were conducted at a rate of 1 cm/min on hydrogel samples with dimensions of 15 mm in diameter and 5 mm in height.

*Swelling Ratio Test.* The hydrogels were placed in 1 mL of PBS and weighed at designated intervals (0, 6, 12, 16, 24, 48, 72, 96, 120, and 144 h), with surface water removed prior to weighing. The swelling ratio was determined using the following formula:

$$\begin{aligned}\:Swelling\:Ratio\:\left(\%\right)\\=\:\frac{Wn-W0}{W0}\:\times\:100\%\end{aligned}$$

where W0 represents the initial weight and Wn represents the weight recorded after n hours.

*pH-responsive degradation*. Buffers with pH 5, 7, 9 were prepared by adjusting the pH of PBS buffer using 0.2 mol/L HCl or NaOH solution. The CSp-OxD@Cu^2+^/G hydrogels were placed in 10 mL of buffers with varying pH levels, taken out at designated intervals (0, 6, 12, 16, 24, 48, 72, 96, 120, and 144 h), freeze-dried, and then weighed. The degradation rate was determined using this method:

$$\begin{aligned}\:Degradation\:Ratio\:\left(\%\right)\\=\:\frac{Wd0-Wdn}{Wd0}\:\times\:100\%\end{aligned}$$

where Wd0 represents the starting dry weight and Wdn represents the dry weight after n hours.

*Cu*^*2+*^ *release test*. The CSp-OxD@Cu^2+^/G hydrogels were placed in 10 mL of buffers with varying pH levels. On days 1, 2, 3, 4, 5, 6, and 7, buffers were taken out to test the Cu^2+^ concentration with the Copper Colorimetric Assay Kit.

## Biocompatibility of CSp-OxD@Cu^2+^/G hydrogel

*Live/Dead Cell Assay*. Cells were treated with Calcein/PI Cell Viability/Cytotoxicity Assay Kit (Beyotime, China) and later observed under fluorescence microscope. The relative fluorescence intensity was calculated using the following formula:

$$\begin{aligned}Relative\; fluorescence\; intesity\\=\frac{Intensity(3d,x)/Intensity(1d,x)}{Intensity(3d,c)/Intensity(1d,c)}\end{aligned}$$

where Intensity (3d, x) and Intensity (1d, x) are the measurements of green fluorescence at 3 days and 1 day in the experimental group, and Intensity (3d, c) and Intensity (1d, c) are the measurements at 3 days and 1 day in the control group.

*Hematoxylin and eosin (H&E).* To assess in vivo biocompatibility of the CSp-OxD@Cu^2+^/G hydrogel, H&E staining was performed on mouse organs (heart, liver, spleen, lung, and kidney) on the 14th day.

*Haemostatic ability*. CSp-OxD@Cu^2+^/G hydrogel’s haemostatic effectiveness was evaluated using a liver hemorrhage model in C57BL/6 mice. The mice were anesthetized with 4 wt% chloral hydrate and positioned on a surgical pad, after which an abdominal incision was made to expose the liver. A filter paper was positioned on a paraffin membrane under the liver. A standard puncture wound was made, and a hemostatic agent (sponge, CSp-OxD hydrogel, or CSp-OxD@Cu^2+^/G) was used. After three minutes, the amount of blood absorbed by the filter paper was measured to evaluate the hemostatic response.

### Cell culture

*HUVEC*. The culture of HUVECs was maintained in a cell incubator at 37 ℃ with 5% CO_2_, using DMEM/F12 medium with an addition of 10% fetal bovine serum (FBS) and 1% penicillin-streptomycin.

*RAW264.7*. The RAW264.7 cells were grown in an incubator set to 37 ℃ with 5% CO_2_, utilizing a specialized RAW264.7 cell culture medium from Servicebio, China.

*CD4*^*+*^ *T cell*. T cells were extracted from the spleens of C57BL/6 mice using a CD4^+^ T cell extraction kit (Beaverbio, China). Briefly, various biotin-tagged monoclonal antibodies were employed to mark non-target cells, which were subsequently removed using streptavidin-coated magnetic beads to sort mouse CD4^+^ T cells.

Th17/Treg induction. To induce Th17 polarization, CD4^+^ T cells need to be treated with the following cytokines. IL-6: 30 ng/ml, TGF-β: 3 ng/ml, TNF-ɑ: 10 ng/ml, IL-1β: 10 ng/ml, anti-IFN-γ: 5 µg/ml, anti-IL-4: 5 µg/ml. To induce Treg polarization, CD4^+^ T cells need to be treated with the following cytokines. IL-2: 5 ng/ml, TGFβ: 3 ng/ml.

*RAW264.7 and CD4*^*+*^*T co-culture*. Co-culture was performed using the Transwell system. RAW264.7 cells were inoculated in the upper chamber and CD4^+^T cells in the lower chamber, and the cells were collected for subsequent experiments after 3 days of co-culture.

### Flow cytometry

*RAW264.7*. After incubating RAW264.7 cells with PE-anti-CD86 antibody at 4 ℃ for 30 min, they were washed with PBS. The cells were then permeabilized and stained with PerCP/Cyanine5.5 anti-CD206 antibody at 4 ℃ for 30 min before being washed again.

*CD4*^*+*^ *T cell*. The CD4^+^ T cells were stained using APC-anti-CD4, PE-anti-IL17 (Th17) and PE-anti-Foxp3 (Treg). Flow cytometry assessed the phenotypes, with analysis conducted using FlowJo software.

### qRT‒PCR analysis

The RNA-easy Isolation kit from Vazyme Biotech, China, was used to extract total RNA, which was then reverse-transcribed using the HiScriptIIIRT SuperMix kit from the same company, adhering to the given instructions. The ChamQSYBR qPCR Master Mix from Vazyme Biotech was used to conduct quantitative reverse transcription polymerase chain reaction (qRT-PCR) on a CFX96 Real-Time PCR Detection System from Bio-Rad, USA. The primers used in this study were sourced from SeqHealth, China, and are detailed as follows:

**Table:** 

	Forward	Reverse
β-actin	GGCTGTATTCCCCTCCATCG	CCAGTTGGTAACAATGCCATGT
TNF-α	GATCGGTCCCCAAAGGGATG	CCACTTGGTGGTTTGTGAGTG
IL-1β	TGCCACCTTTTGACAGTGATG	TGATGTGCTGCTGCGAGATT
iNOS	TCTAGTGAAGCAAAGCCCAACA	CCTCACATACTGTGGACGGG
Arg1	GTAGACCCTGGGGAACACTAT	ATCACCTTGCCAATCCCCAG
IL-4	CTCACAGCAACGAAGAACACC	CTGCAGCTCCATGAGAACACT
IL-10	GCTGTCATCGATTTCTCCCCT	GACACCTTGGTCTTGGAGCTTAT
PDK1	AGGATTACTTTATAGACCGGGTCAG	AGGCAACTCTTGTCGCAGAA
PKM2	GCAGCGACTCGTCTTCACTT	ATGGTTCCTGAAGTCCTCGG
LDHA	AGTAAGTCCTCAGGCGGCTA	GGACTTTGAATCTTTTGAGACCTTG
PFK1	GGAGAGCTAAAACTACAAGAGTGG	CTCCACCAGAGGTCAACACG
HK2	GTTTCTCTATTTGGCCCCGAC	AGAGATACTGGTCAACCTTCTGC
GLUT1	ATAGTTACAGCGCGTCCGTT	GGCGGTGTTCATCGGTCTTG
IL2	TGCGGCATGTTCTGGATTTG	CAAATGTGTTGTCAGAGCCCTTT
TGF-β	ACGTTCCCAAGTCGGATGTG	TTCAGTGGATGGATGGTCCT
IL-17	CTGGACTCTCCACCGCAATG	GGACCAGGATCTCTTGCTGG
IL-21	GCCCATAAATCAAGCCCCCA	GCTCACAGTGCCCCTTTACA

### Western blotting analysis

After resolving protein extracts from different groups with 10% SDS-polyacrylamide gel electrophoresis (SDS-PAGE), they were transferred to PVDF membranes. The membranes were incubated with 5% bovine serum albumin (BSA) for one hour, then exposed overnight at 4 ℃ to primary antibodies specific for β-actin (1:4000), NLRP3 (1:2000), GSDMD (1:1000), Caspase 1 (1:1000), and IL-1β (1:1000). Subsequently, the membranes were rinsed with Tris-buffered saline with Tween-20 (TBS-T) and then exposed to horseradish peroxidase (HRP)-linked secondary antibodies for an hour at room temperature. Protein bands were visualized using an enhanced chemiluminescence detection system from Beyotime Biotechnology, China.

### Mitochondrial membrane potential assay

The Mitochondrial Membrane Potential Assay Kit (Beyotime, China) was utilized to perform the assay following this protocol: First, the culture medium was aspirated, and the cells were washed with PBS. Next, the cells were incubated with 1 mL of culture medium and 1 mL of JC-1 staining solution at 37 ℃ for 20 min. Post-incubation, the supernatant was eliminated, and the cells were washed twice with a cold JC-1 staining buffer. Finally, 2 mL of culture medium was added back to the cells for subsequent observation using fluorescence microscopy.

### Glycolysis/oxidative phosphorylation assay

The Glycolysis/OXPHOS Assay Kit from Dojindo, Japan, was utilized to measure lactate and ATP levels, as well as to assess the extent of cellular reliance on glycolysis and oxidative phosphorylation pathways.

### Untargeted metabolomics assays and analysis

*Sample preparation and extraction*. The sample, previously stored at −80 ℃, was thawed on ice. Subsequently, a 500 µL solution consisting of methanol and water in a 4:1 volume ratio, containing an internal standard, was added to the cell sample and vortexed for 3 min. The sample was then subjected to liquid nitrogen for 5 min, followed by exposure to dry ice for an additional 5 min. After this, it was thawed on ice and vortexed for 2 min. This freeze-thaw cycle was performed a total of three times. The sample underwent centrifugation at 12,000 rpm for 10 min at 4 °C, following which 300 µL of the supernatant was extracted and stored at −20 °C for half an hour. The sample was then centrifuged once more at 12,000 rpm for 3 min at 4 ℃, and 200 µL portions of the supernatant were set aside for LC-MS analysis.

*Analytical Methods*. All analyses were completed on the Cloud Platform of Metware, China.

### Diabetic wound model

C57BL/6 mice (6 week) were sourced from Hunan SJA Laboratory Animal Co., Ltd (China) and utilized with the approval of the Animal Research Ethics Committee of Tongji Medical College, Huazhong University of Science and Technology.

Diabetes was induced by feeding mice a high-fat diet for four weeks, followed by daily intraperitoneal injections of streptozotocin (STZ, 50 mg/kg, Sigma, USA) for three days in a row. Diabetes was identified based on fasting blood glucose levels that were consistently higher than 11.1 mmol/L across three tests.

*Skin wound modeling and healing*. Mice were anesthetized with sodium pentobarbital at a dosage of 50 mg/kg. Full-thickness dorsal wounds, measuring 15 mm in diameter, were created using a sharp round punch. Various treatment modalities were administered on days 0, 3, 7, and 10 following the wounding procedure. Photographic documentation of the wounds was conducted and subsequently analyzed using ImageJ software on days 0, 3, 7, 10, and 14. The ratio of the remaining wound area was calculated according to the following methodology:

$$\begin{aligned}\:Wound\:Remaining\:ratio\:\left(\%\right)\\=\:\frac{S0-Sn}{S0}\:\times\:100\%\end{aligned}$$

with S0 denotes the wound area on day 0 and Sn denotes the wound area on day n.

### Histologic examination

*H&E and masson staining*. Hematoxylin and eosin (H&E) and Masson’s trichrome (MT) staining were conducted to evaluate wound sites at day 14.

*Immunofluorescence staining*. On the seventh day, macrophage polarization in wounds was indicated by the assessment of M1 and M2 macrophages using CD86 and CD206. CD4 and IL-17 were used to assess Th17 at day 7; CD4 and Foxp3 were used to assess Treg also at day 7. Using an Olympus IX53 microscope, images were captured and analyzed with the help of ImageJ software.

### Statistical analysis

All data analyses were conducted using GraphPad Prism version 9.0 software, with a p-value threshold of less than 0.05 established to indicate statistical significance. Mean values with standard deviation (SD) are used to present the results. Comparisons between two groups were made using Student’s t-tests. In cases where multiple groups were analyzed, a one-way ANOVA was performed, followed by the application of the Benjamini-Hochberg procedure to adjust for false discovery rates. The sample size for each group was a minimum of three.

## Results

### Synthesis and characterization of Cu^2+^/G NPs

**Fig. 2:**
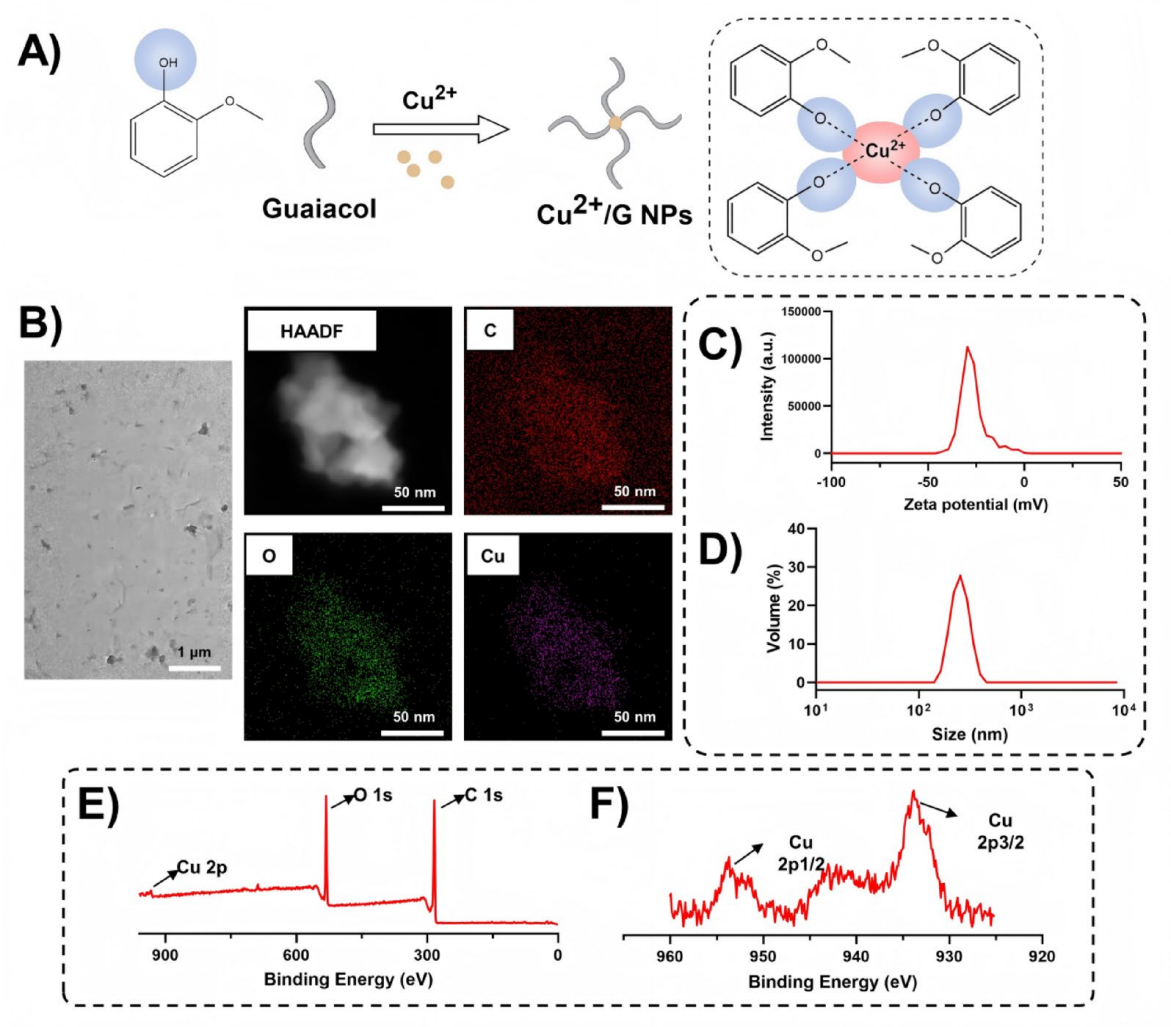
Synthesis and analysis of Cu^2+^/G nanoparticles. (**A**) Schematic diagram of CSp-OxD@Cu^2+^/G hydrogel formation; (**B**) Transmission electron microscope (TEM) and elemental mapping, (**C-D**) Zeta potential and particle size, (**E-F**) X-ray photoelectron spectroscopy (XPS) results of Cu^2+^/G nanoparticles

A schematic representation of the CSp-OxD@Cu^2+^/G hydrogel system’s construction is depicted in Fig. 2A. The primary raw materials utilized for synthesizing the CSp-OxD dual-crosslinked hydrogel were chitosan and dextran. These were subjected to 3-carboxyphenylboronic acid (3-CPBA) grafting and NaIO_4_ oxidation, respectively, resulting in the formation of CPBA-grafted chitosan (CSp) and oxidized dextran (OxD). The boronic acid moiety and amino groups of CSp reacted with the hydroxyl and aldehyde groups of OxD, forming borate and Schiff base linkages, respectively, thereby endowing the CSp-OxD hydrogel with a dual-crosslinked structure. The borate linkage exhibits pH-responsive behavior, dissociating in the presence of H^+^, which facilitates controlled drug release in the mildly acidic environment characteristic of diabetic wounds. Conversely, the Schiff base linkage serves to maintain the fundamental structural integrity of the hydrogel system, compensating for the loss of physical support properties upon borate bond dissociation.

As illustrated in Fig. 2B, the particle size distribution of the Cu^2+^/guaiacol nanoparticles (Cu^2+^/G NPs) was predominantly uniform, with no significant agglomeration observed. Elemental mapping further confirmed the homogeneous distribution of carbon (C), nitrogen (N), and copper (Cu) elements within the Cu^2+^/G NPs. Dynamic Light Scattering (DLS) experiments were conducted to assess the zeta potential and particle size of Cu^2+^/G NPs at pH = 7 (Fig. 2C, D). The findings indicated that the particle sizes of Cu^2+^/G NPs predominantly ranged from 150 nm to 400 nm, with an average diameter of 323 nm. The Cu^2+^/G NPs exhibited a negative charge, with an average absolute zeta potential value of 26.8 mV. This substantial zeta potential suggests that the Cu^2+^/G NPs possess significant electrostatic repulsion, reducing the likelihood of agglomeration and promoting a homogeneous dispersion, consistent with the results obtained from transmission electron microscopy (TEM) experiments [37, 38]. At pH levels of 5 and 9, the Cu^2+^/G NPs displayed zeta potentials of −15.9 mV and − 32.3 mV, respectively, and average particle sizes of 386 nm and 342 nm, respectively. These observations imply that the nanoparticles can readily achieve a uniformly dispersed state in both weakly acidic and weakly alkaline conditions, rendering them suitable for physiological environments (Figure S1A-D). The negative charge of Cu^2+^/G NPs primarily originates from phenolate anions. The phenolic hydroxyl group (-OH) in guaiacol undergoes deprotonation, losing a hydrogen ion (H^+^) to form a phenolate anion (-O^−^). Correspondingly, the deprotonation capability of the phenolic hydroxyl group is weakened in a weakly acidic environment, leading to a decrease in the absolute value of the zeta potential of Cu^2+^/G NPs, which aligns with the expected outcome. Additionally, the presence of C 1 s, O 1 s, and Cu 2p signals in the X-ray photoelectron spectra (XPS) further corroborates the successful synthesis of Cu^2+^/G NPs (Fig. 2E, F).

### Synthesis and characterization of CSp-OxD@Cu^2+^/G hydrogel

**Fig. 3:**
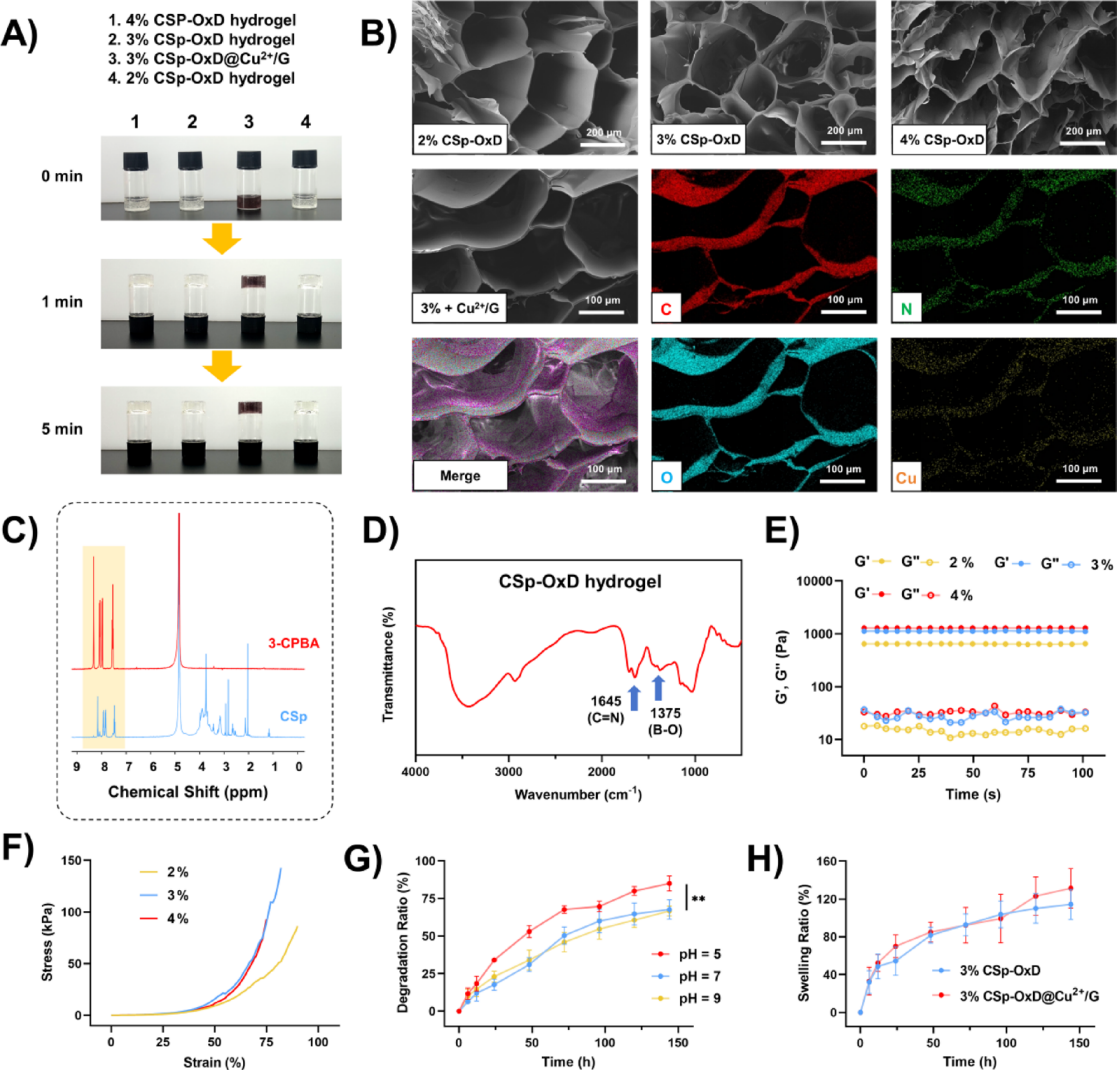
Synthesis and Characterization of CSp-OxD@Cu^2+^/G Hydrogel. **A**) Macroscopic visualization of the synthesis process of the CSp-OxD@Cu^2+^/G hydrogel; **B**) Representative scanning electron microscopy (SEM) and energy dispersive spectrometer (EDS) images of CSp-OxD hydrogel and CSp-OxD@Cu^2+^/G hydrogel at varying concentrations; **C**) Nuclear magnetic resonance (NMR) hydrogen spectra of 3-CPBA and CSp; D) Fourier-transform infrared spectroscopy (FTIR) results of CSp-OxD hydrogel; **E-F**) Rheological and compression test results of CSp-OxD hydrogels at different concentrations; **G**) Degradation characteristics of CSp-OxD hydrogel under various pH conditions; **H**) Swelling rates of CSp-OxD hydrogel and CSp-OxD@Cu^2+^/G hydrogel. Data are presented as mean ± standard deviation (SD), *n* = 3; *P < 0.05, **P < 0.01, ***P < 0.001, and ns, not significant

To facilitate the efficient delivery of Cu^2+^/G NPs, we developed an injectable, hemostatic dual-crosslinked CSp-OxD hydrogel. CSp was synthesized by grafting 3-carboxyphenylboronic acid onto chitosan, while OxD was prepared through the oxidation of dextran with NaIO_4_. The elemental compositions of CSp and OxD were determined using an elemental analyzer (Table S1). Based on the comparison and calculation of C/N (O/N, H/N) ratios, the degree of substitution of 3-CPBA in CSp was determined to be 34.9% (34.8%, 34.7%). From the comparison and calculation of C/O (C/H) ratios, the oxidation degree of dextran was found to be 46.1% (44.8%). Figure 3A illustrates the macroscopic examination of the hydrogel formation process at varying concentrations, specifically 2%, 3%, and 4% CSp-OxD hydrogels, as well as CSp-OxD@Cu^2+^/G hydrogels. These hydrogels rapidly form and maintain their structural integrity without significant deformation within 5 min of inversion, demonstrating solid-state characteristics. This observation indicates that the hydrogel system has developed an effective three-dimensional cross-linked network with adequate structural stability, which remains unaffected by the presence of Cu^2+^/G NPs.

**Fig. 4:**
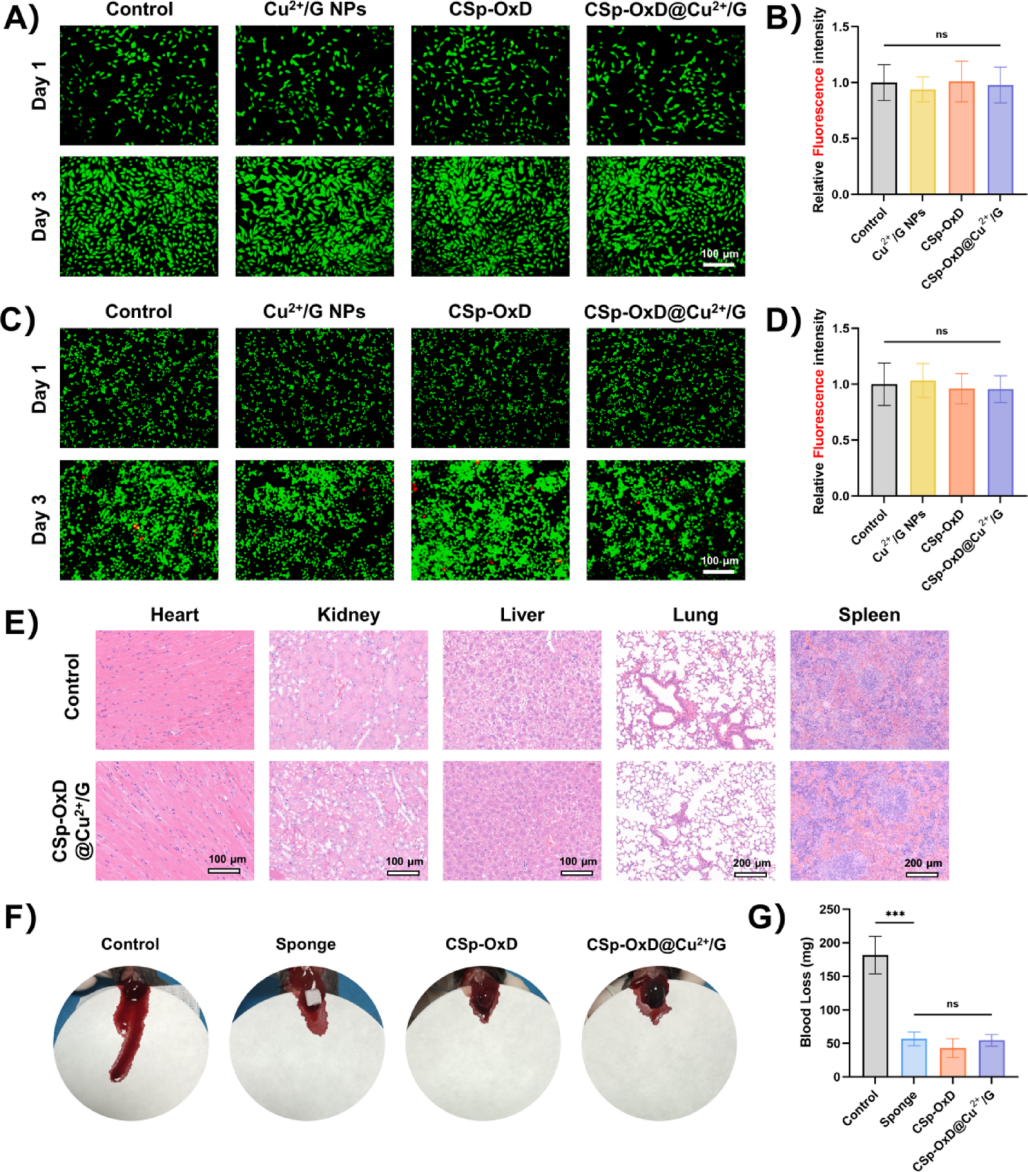
Biocompatibility and hemostatic properties of CSp-OxD@Cu^2+^/G hydrogel. A-B) Live/dead staining images and statistics for *HUVEC*s across treatment groups; C-D) Live/dead staining images and statistics for *RAW264.7* cells across treatment groups; E) H&E staining of mouse heart, kidney, liver, lung, and spleen across treatment groups; F-G) Hemostatic capacity and blood loss data for CSp-OxD@Cu^2+^/G hydrogel. Data are presented as mean ± SD, *n* = 3; *P < 0.05, **P < 0.01, ***P < 0.001, and ns, not significant

To further investigate the microstructure and elemental composition of the hydrogels, scanning electron microscopy (SEM) and energy-dispersive spectrometry (EDS) were employed. These techniques were combined to analyze the spatial distribution of various elements within the hydrogel system [39–41]. As depicted in Fig. 3B, CSp-OxD hydrogels display a loose, porous microstructure, and the distribution of copper elements suggests a relatively uniform dispersion of Cu^2+^/G NPs within the CSp-OxD hydrogel. Additionally, nuclear magnetic resonance (NMR) hydrogen spectroscopy and Fourier-transform infrared (FTIR) spectroscopy were utilized to characterize the intermolecular cross-linking state [42]. As illustrated in Figs. 3C and 4-CPBA exhibits resonances at 8.4, 8.1, 8.0, and 7.6 ppm, indicative of a benzene ring structure. CSp displays similar benzene ring resonances, thereby confirming the successful grafting of 3-CPBA. Fourier transform infrared spectroscopy (FTIR) serves as a tool to elucidate functional groups and chemical bonds within a sample and is employed to assess whether the cross-linking status of the hydrogel system aligns with anticipated outcomes. In the CSp-OxD hydrogel, the peak observed at 1645 cm^− 1^ signifies the presence of a carbon-nitrogen double bond, corroborating the formation of a Schiff base, while the peak at 1375 cm^− 1^ signifies a boron-oxygen single bond, confirming the formation of a borate ester bond (Fig. 3D). The FTIR results substantiate that the dual-crosslinked structure of CSp-OxD hydrogels is constituted by Schiff bases and borate ester bonds, in accordance with expectations.

Subsequently, rheological assessments and compression tests were conducted to evaluate the physical properties of CSp-OxD hydrogels across varying concentrations, facilitating the selection of the optimal hydrogel concentration. As depicted in Fig. 3E, the rheological time sweep analysis reveals that both the storage modulus (G’) and the loss modulus (G”) of CSp-OxD hydrogels exhibit an increase with rising concentration levels. These hydrogel systems, across various concentrations, consistently demonstrate viscoelastic properties, thereby indirectly indicating the injectability of CSp-OxD hydrogels. Meanwhile, CSp-OxD hydrogel was loaded into a 1mL syringe for pattern drawing to demonstrate its injectability (Figure S2). In compression testing, the 3% CSp-OxD hydrogel successfully withstands pressures nearing 150 kPa and endures 80% deformation without structural failure, thereby exhibiting superior compressive strength (Fig. 3F). Consequently, the 3% CSp-OxD hydrogel was selected for subsequent experimental procedures. Meanwhile, the incorporation of Cu^2+^/G NPs did not affect the rheological or compressive properties of the CSp-OxD hydrogel (Figure S12, S13). The step-strain test results confirmed the injectability of the hydrogel (Figure S14). The self-healing behavior of the CSp-OxD hydrogel is demonstrated in Figure S15.

Given the pH-responsive nature of borate ester bonds, we examined the degradation behavior of CSp-OxD hydrogels under varying pH conditions (pH = 5, 7, and 9). The hydrogels displayed rapid degradation at pH = 5, with degradation rates reaching 68% and 85% on day 3 and 6, respectively. Conversely, the degradation rate was markedly slower in neutral and mildly alkaline environments, achieving approximately 67% by day 6 (Fig. 3G). Subsequently, the hydrogel system was subcutaneously implanted in the dorsal region of mice to investigate its in vivo degradation behavior. The CSp-OxD@Cu^2+^/G hydrogel retained approximately 30% of its mass by day 3 and was almost completely degraded by day 5 (Figure S11). By monitoring the concentration of free Cu^2+^, we investigated the dissociation characteristics of Cu^2+^/G NPs loaded within the CSp-OxD hydrogel under different pH conditions. Compared to the neutral environment (pH = 7), a higher Cu^2+^ concentration was observed at pH = 5, indicating that the CSp-OxD@Cu^2+^/G hydrogel releases more active components under weakly acidic conditions, which is consistent with the expected results (Figure S10). The swelling behavior of the hydrogels, indicative of their hydrophilicity, is a critical factor for drug loading applications. As illustrated in Fig. 3H, the hydrogel system exhibited rapid swelling within the first day and achieved equilibrium by day 6. Notably, the presence of Cu^2+^/G NPs did not significantly influence the swelling behavior of the CSp-OxD hydrogel.

### Biocompatibility and hemostatic properties of CSp-OxD@Cu^2+^/G hydrogel

The CSp-OxD@Cu^2+^/G hydrogel demonstrates excellent biocompatibility and hemostatic capability. The in vitro biocompatibility of the CSp-OxD@Cu^2+^/G hydrogel was confirmed through live/dead staining assays. Given the direct involvement of endothelial cells in the wound healing process and the critical role of macrophages as key inflammatory regulatory cells, *HUVEC*s and *RAW264.7* cells were selected to assess the biocompatibility of the CSp-OxD@Cu^2+^/G hydrogel. As illustrated in Fig. 4A, B, there was no statistically significant difference in the live/dead cell ratio of *HUVEC*s following various treatments. Figure 4C, D demonstrate comparable live/dead staining results for *RAW264.7* cells. The CCK8 assay further proved that the CSp-OxD@Cu^2+^/G hydrogel exhibited excellent biocompatibility (Figure S3A-C). Subsequent hematoxylin and eosin (H&E) staining of tissue samples indicated no discernible abnormalities in the organs of mice treated with the CSp-OxD@Cu^2+^/G hydrogel (Fig. 4E). The hemostatic efficacy of the hydrogel system was evaluated using a mouse liver hemorrhage model. Upon comparing the blood loss across different treatment groups, the CSp-OxD@Cu^2+^/G hydrogel exhibited a hemostatic effect comparable to that of the positive control (hemostatic sponge) (Fig. 4F). The hemostatic capability of the CSp-OxD@Cu^2+^/G hydrogel originates from its rapidly established physical barrier and multiple biological interactions with blood components. Upon contact with the wound, the soft hydrogel immediately fills tissue interstices and forms an in-situ gel through dynamic covalent cross-linking, effectively sealing damaged blood vessels. Simultaneously, the positively charged chitosan chains strongly adsorb negatively charged red blood cells and platelets via electrostatic interactions, while the active aldehyde groups of oxidized dextran directly activate platelets, promoting their aggregation and release. Together, these mechanisms significantly accelerate the formation of the primary hemostatic plug and the initiation of the coagulation cascade.

### CSp-OxD@Cu^2+^/G hydrogel recovers macrophage polarization

**Fig. 5:**
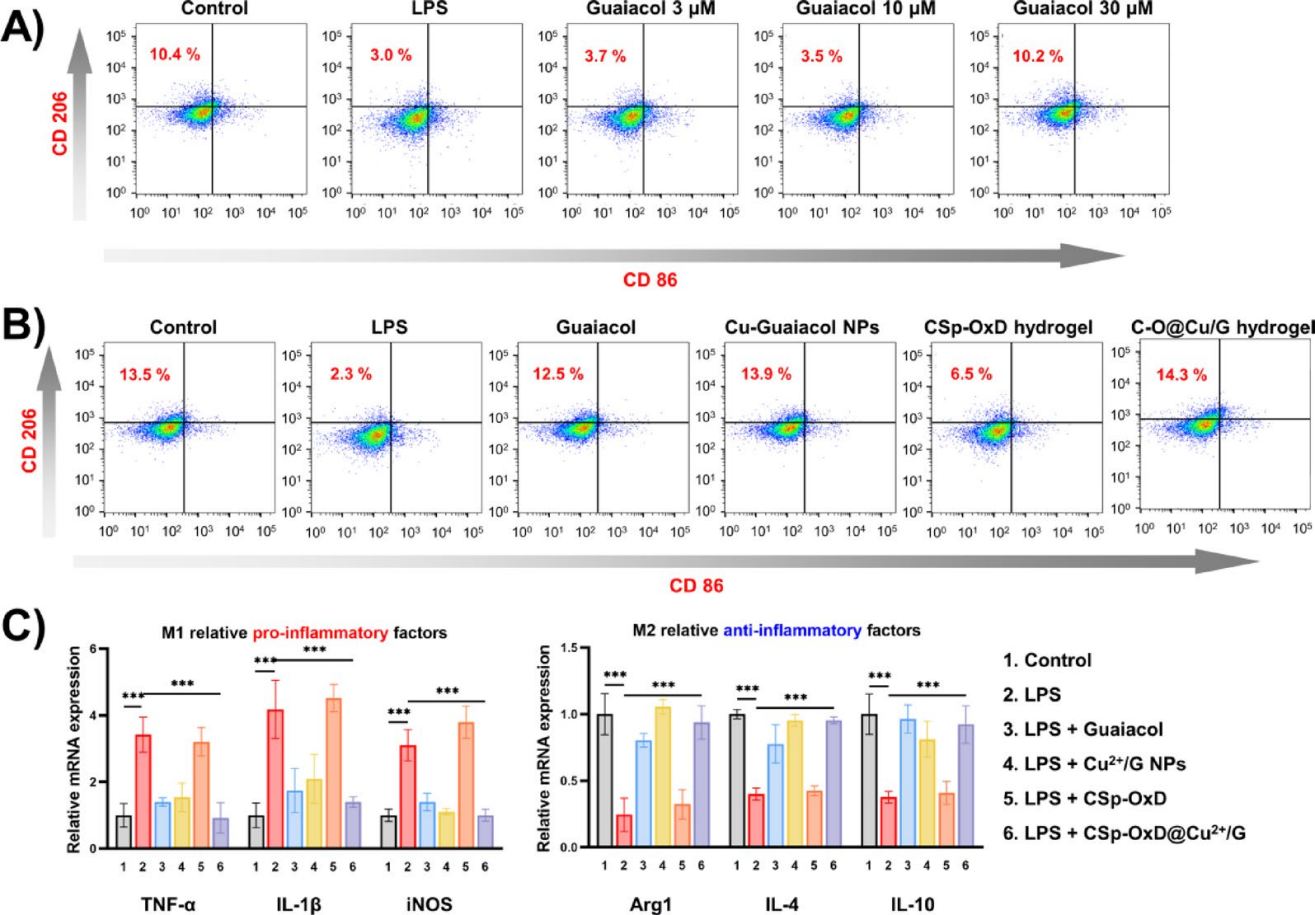
CSp-OxD@Cu^2+^/G hydrogel influences macrophage polarization and inflammatory factor expression. **A**) Effect of different concentrations of guaiacol on *RAW264.7* cell polarization; Hydrogel’s role in regulating **B**) *RAW264.7* cell polarization and C) inflammatory factor expression. Data are presented as mean ± SD, *n* = 3; *P < 0.05, **P < 0.01, ***P < 0.001, and ns, not significant

The CSp-OxD@Cu^2+^/G hydrogel demonstrated anti-inflammatory properties by modulating macrophage polarization towards the M2 phenotype and altering the expression of inflammatory factors. Flow cytometry was employed to analyze the cell typing of *RAW264.7*. Various concentrations of guaiacol were administered to macrophages to ascertain the optimal drug concentration (Fig. 5A, S4A). It was found that 30 µM guaiacol effectively reversed lipopolysaccharide (LPS)-induced M1 polarization of macrophages without exhibiting cytotoxicity. As illustrated in Fig. 5B and S4B, the M2 polarization ratio in the Cu^2+^/G NPs group was comparable to that observed in the guaiacol group, suggesting that chelation with CuCl_2_ does not significantly alter the anti-inflammatory properties of guaiacol. The CSp-OxD@Cu^2+^/G hydrogel group did not show a higher proportion of M2-polarized macrophages, indicating that the hydrogel system neither enhanced nor compromised the drug’s efficacy. The experimental findings regarding macrophage inflammatory factor expression were consistent with the flow cytometry results. As depicted in Fig. 5C, treatment with the CSp-OxD@Cu^2+^/G hydrogel resulted in a reduction in the expression of pro-inflammatory factors (TNF-ɑ, IL-1β, iNOS) and an increase in the expression of anti-inflammatory factors (Arg1, IL-4, IL-10) in *RAW264.7* cells.

#### CSp-OxD@Cu^2+^/G hydrogel reprograms macrophage pyroptosis and metabolism

Previous experiments have demonstrated that CSp-OxD@Cu^2+^/G hydrogels can induce macrophages to adopt an anti-inflammatory phenotype. To further elucidate the regulatory mechanisms governing macrophage function, future investigations will concentrate on pyroptosis and the reprogramming of energy metabolism. Pyroptosis, a pro-inflammatory form of cell death, significantly impacts the local inflammatory milieu through its occurrence and intensity [43, 44]. Concurrently, the state of energy metabolism is pivotal in determining the differentiation pathways and functional outcomes of macrophages [45, 46]. Investigating these interconnected processes will yield critical insights into the mechanisms that regulate macrophage function. During pyroptosis, proteins such as NLRP3, cleaved-caspase-1, cleaved-IL-1β, and cleaved-GSDMD serve as essential molecular markers, with their dynamic changes directly reflecting the activation status and regulatory mechanisms of the pyroptosis pathway [47, 48].

**Fig. 6:**
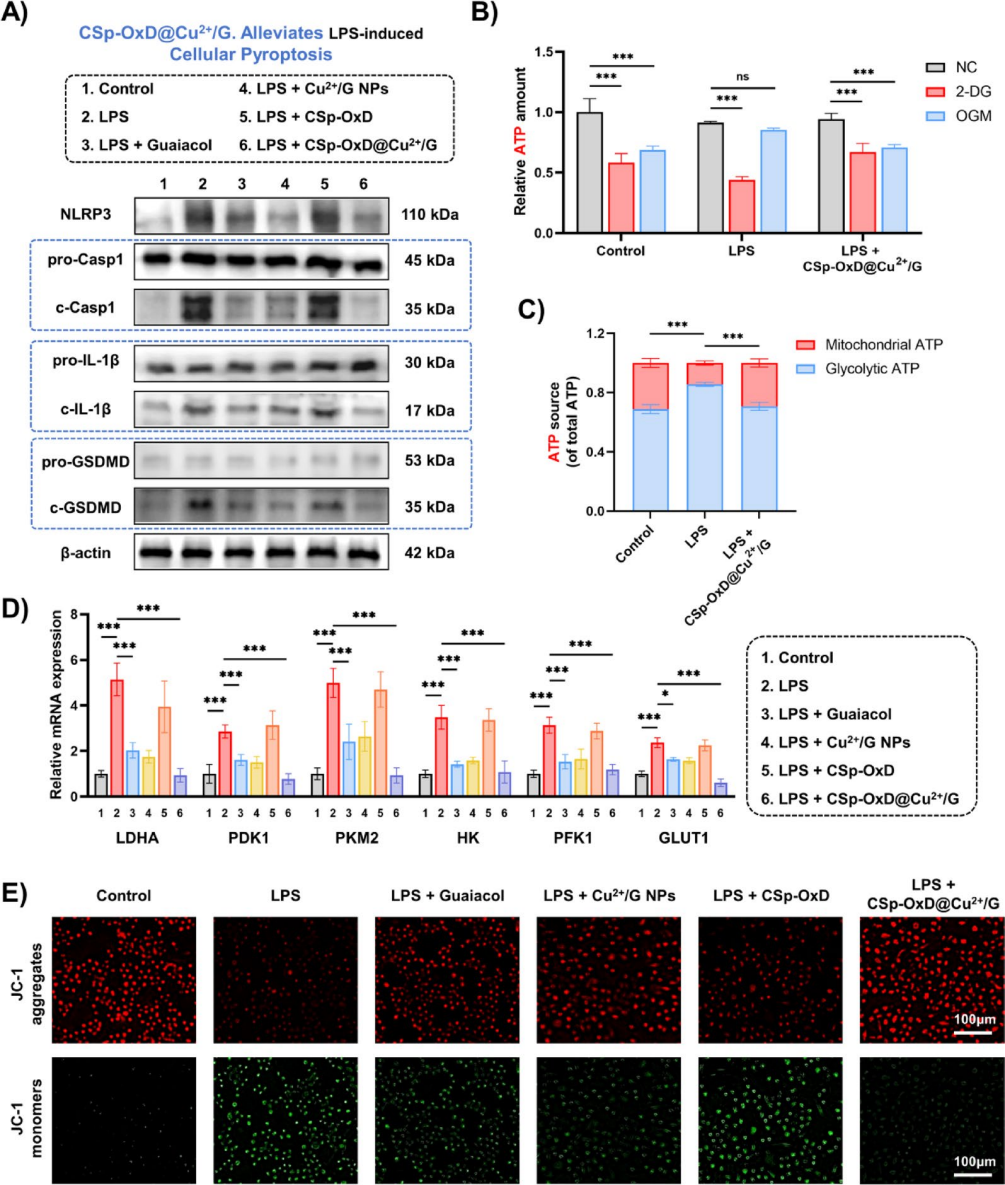
CSp-OxD@Cu^2+^/G hydrogel affects macrophage pyroptosis and energy metabolism. **A**) Western blot analysis, **B**) Relative ATP levels, **C**) ATP source proportions, **D**) Glycolysis-related mRNA expression and **E**) JC-1 staining results of *RAW264.7* under various treatments. Data are presented as mean ± SD, *n* = 3; *P < 0.05, **P < 0.01, ***P < 0.001, and ns, not significant

Western blot analyses have indicated that the expression levels of these core molecular markers increase following LPS treatment, whereas the expression of precursor fragments (pro-caspase-1, pro-IL1β, pro-GSDMD) remains unaltered (Fig. 6A). This study illustrates that LPS markedly amplifies the pyroptosis process in *RAW264.7*. The CSp-OxD@Cu^2+^/G hydrogel system mitigates the effects induced by LPS, as evidenced by a substantial reduction in the expression of key molecular markers in the LPS + CSp-OxD@Cu^2+^/G group compared to the LPS group, aligning closely with the levels observed in the Control group. Furthermore, no significant differences were observed in the expression of core molecular markers between the LPS + Guaiacol group and the LPS + Cu^2+^/G NPs group, further indicating that chelation with CuCl_2_ does not alter the pharmacological efficacy of guaiacol.

Through the application of ATP content detection, JC-1 fluorescent probe analysis, and qRT-PCR experiments, it has been demonstrated that the CSp-OxD@Cu^2+^/G hydrogel system effectively reprograms the energy metabolism of macrophages. As illustrated in Figs. 6B, C, the CSp-OxD@Cu^2+^/G hydrogel mitigates the increased glycolytic activity and reliance on the glycolytic pathway observed in *RAW264.7* cells under the influence of LPS. The ATP content in *RAW264.7* was assessed under various treatment conditions (Control, LPS, LPS + CSp-OxD@Cu^2+^/G), with Oligomycin (OGM) and 2-deoxy-D-glucose (2-DG) employed to inhibit ATP synthesis via oxidative phosphorylation and glycolysis pathways, respectively. As depicted in Fig. 6B, in the presence of 2-DG, the ATP content of *RAW264.7* in the LPS group was reduced compared to the Control group; conversely, following the addition of OGM, the ATP content in the LPS group exceeded that of the Control group. These findings indicate that LPS treatment enhances glycolytic activity and dependence on glycolysis in macrophages. Notably, the CSp-OxD@Cu^2+^/G hydrogel system is capable of reversing the effects induced by LPS, restoring the elevated glycolytic activity to levels comparable to the Control group, as shown in Fig. 6C. Quantitative real-time PCR (qRT-PCR) experiments were performed to analyze the expression levels of mRNA associated with glycolysis, thereby corroborating the regulatory influence of the CSp-OxD@Cu^2+^/G hydrogel system on the glycolytic activity of macrophages (Fig. 6D).

Furthermore, given that a high mitochondrial membrane potential is fundamental to energy production within cellular metabolism, the JC-1 fluorescent probe was employed to assess mitochondrial membrane potential. In conditions of elevated mitochondrial membrane potential, JC-1 aggregates within the mitochondrial matrix, forming J-aggregates that emit red fluorescence; conversely, under conditions of reduced membrane potential, JC-1 remains in its monomeric form, emitting green fluorescence [49, 50]. As illustrated in Fig. 6E, following LPS treatment, a marked reduction in red fluorescence and an increase in green fluorescence were observed in *RAW264.7* cells, indicating a decline in membrane potential. However, upon treatment with the CSp-OxD@Cu^2+^/G hydrogel, *RAW264.7* cells exhibited pronounced red fluorescence signals comparable to those in the control group, suggesting that the hydrogel system effectively mitigates LPS-induced impairment of macrophage membrane potential. These studies collectively illustrate the therapeutic potential of the CSp-OxD@Cu^2+^/G hydrogel in modulating the pyroptosis pathway and energy metabolism of macrophages.

### CSp-OxD@Cu^2+^/G hydrogel indirectly regulates Th17/Treg balance

**Fig. 7:**
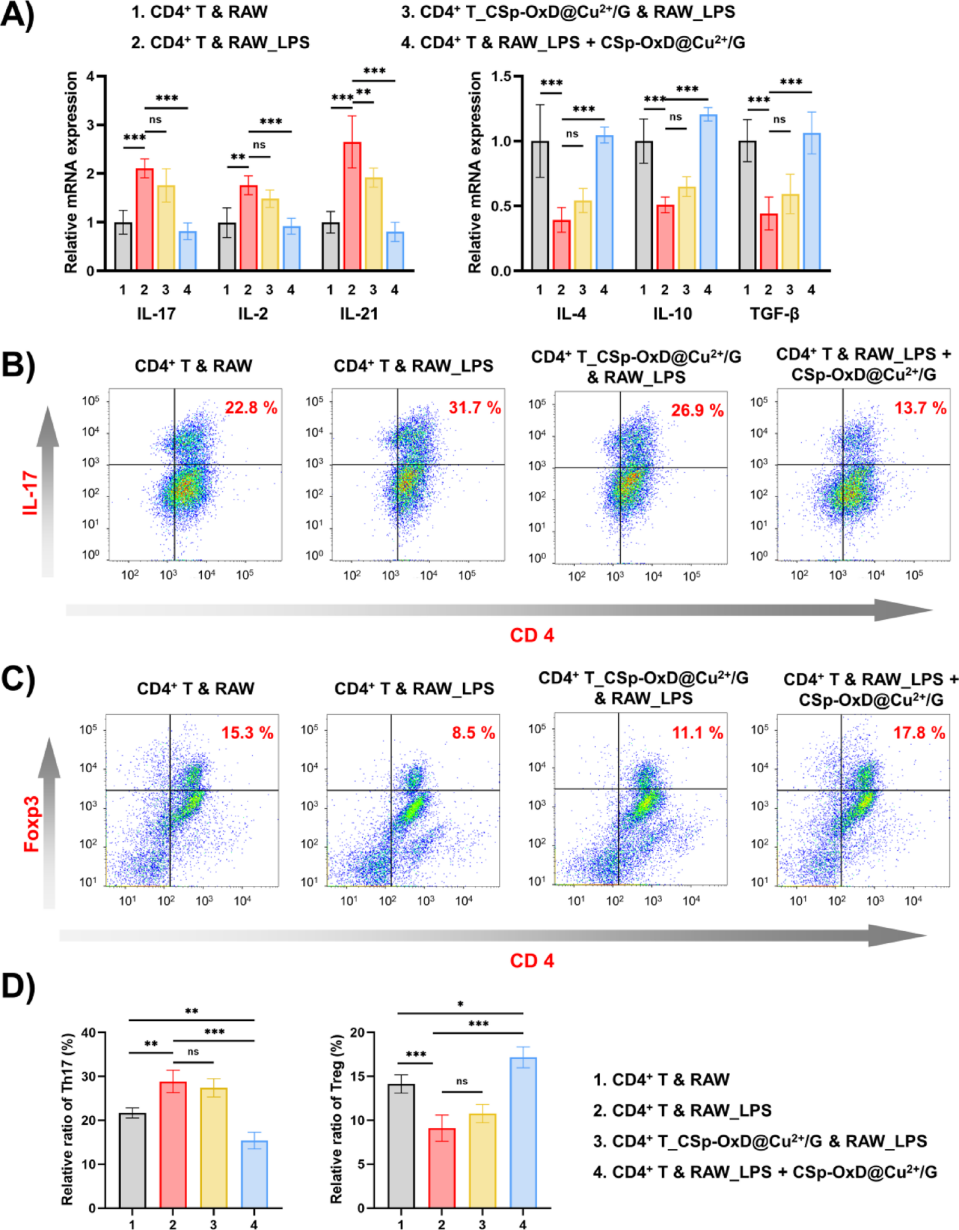
CSp-OxD@Cu^2+^/G hydrogel indirectly regulates Th17/Treg balancing in *CD4*^*+*^ *T* cells. **A**) qRT-PCR results of inflammatory factors in mouse *CD4*^*+*^ *T* cells across different groups; **B-D**) Flow cytometry results and analysis for *CD4*^*+*^ *T* cells across treatment groups. Data are presented as mean ± SD, *n* = 3; *P < 0.05, **P < 0.01, ***P < 0.001, and ns, not significant

The aforementioned experiments have confirmed that CSp-OxD@Cu^2+^/G facilitates the polarization of macrophages towards an anti-inflammatory phenotype, such as the M2 type, thereby effectively mitigating excessive inflammatory responses at the wound site and establishing a conducive microenvironment for tissue regeneration. Nevertheless, wound healing is a complex, highly coordinated immune cascade process that necessitates precise collaboration between innate and adaptive immunity [51]. Within this context, *CD4*^*+*^ *T* lymphocytes, as pivotal effector cells of adaptive immunity, play a critical regulatory role in the later stages of healing [52]. Consequently, we aimed to further explore whether this hydrogel system also exerts regulatory effects on *CD4*^*+*^ *T* cells, specifically the Th17/Treg balancing. As depicted in Fig. 7A, qRT-PCR experiments were employed to assess the expression of inflammatory factors in *CD4*^*+*^ *T* cells. Group 1 served as the untreated control; group 2 comprised LPS-treated *RAW264.7* cells co-cultured with *CD4*^*+*^ *T* cells; in group 3, LPS-treated *RAW264.7* cells were co-cultured with CSp-OxD@Cu^2+^/G-pretreated *CD4*^*+*^ *T* cells; group 4 consisted of LPS-treated *RAW264.7* cells pretreated with CSp-OxD@Cu^2+^/G, followed by co-culture with *CD4*^*+*^ *T* cells. In comparison to group 1, group 2’s mice demonstrated an upregulation of proinflammatory factors and a downregulation of anti-inflammatory factors in splenic *CD4*^*+*^ *T* cells. This suggests that LPS can indirectly modulate *CD4*^*+*^ *T* cell differentiation and function by influencing macrophage polarization, a conclusion corroborated by multiple studies [53, 54]. Notably, our findings indicate that CSp-OxD@Cu^2+^/G does not exert a direct effect on *CD4*^*+*^ *T* cells; rather, it significantly impacts their polarization status by modulating macrophages. As illustrated in Fig. 7A, *CD4*^*+*^ *T* cells in group 3 did not exhibit significant differences in the expression of most inflammatory factors compared to group 2, except for IL-21. Conversely, *CD4*^*+*^ *T* cells in group 4 showed a pronounced shift towards anti-inflammatory activity relative to group 2.

Figures 7B, C further elucidate, through flow cytometry analyses, that the CSp-OxD@Cu^2+^/G hydrogel influences the Th17/Treg balancing of *CD4*^*+*^ *T* cells via macrophage mediation. Statistical analyses reveal that *RAW264.7* cells treated with LPS exhibit an increased proportion of Th17 cells and a diminished proportion of Treg cells within the *CD4*^*+*^ *T* cell population (Fig. 7D). Notably, the CSp-OxD@Cu^2+^/G hydrogel can counteract this effect by modulating macrophages, thereby indirectly restoring the Th17/Treg balance in *CD4*^*+*^ *T* cells.

### Metabolomics analysis of CSp-OxD@Cu^2+^/G hydrogel treated macrophages

**Fig. 8:**
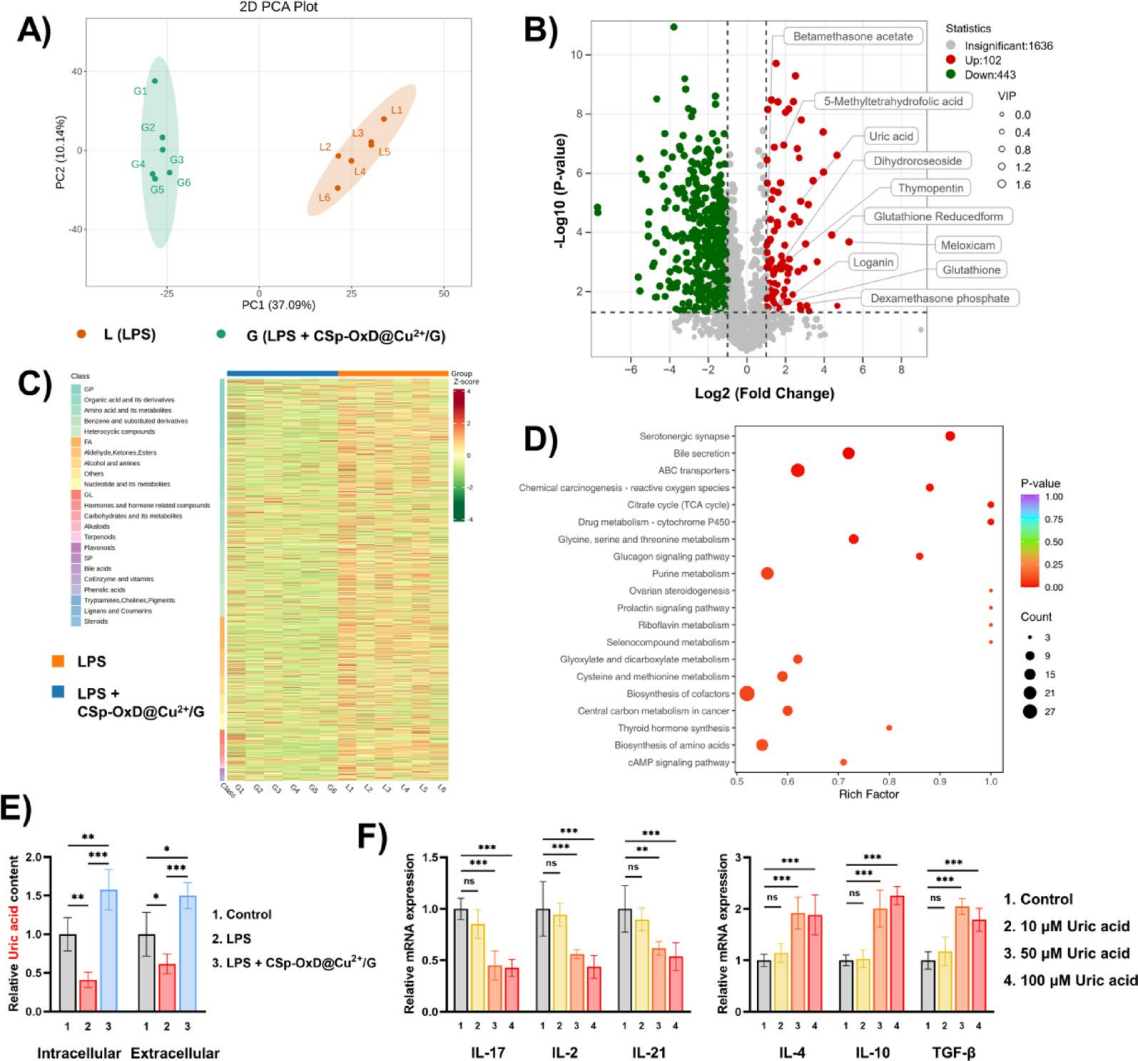
Metabolic effects of CSp-OxD@Cu^2+^/G hydrogel on macrophages. **A-D**) Metabolomics analysis of *RAW264.7* cells (LPS, LPS + CSp-OxD@Cu^2+^/G), including **A**) principal component analysis, **B)** volcano plot of differential metabolites, **C**) clustering heatmap of metabolites, and **D**) KEGG enrichment analysis; **E**) Relative intracellular and extracellular uric acid levels in *RAW264.7* cells under different treatments; **F**) *CD4*^*+*^ *T* cell inflammatory factor expression after varying uric acid concentrations. Data are presented as mean ± SD, *n* = 6; *P < 0.05, **P < 0.01, ***P < 0.001, and ns, not significant

The above observation prompted further investigation. We employed non-targeted metabolomics to assess metabolite levels in *RAW264.7* cells subjected to different treatments (LPS, LPS + CSp-OxD@Cu^2+^/G), with the objective of elucidating the novel regulatory axis through which the CSp-OxD@Cu^2+^/G hydrogel modulates *CD4*^*+*^ *T* cell activity. While it is established that large-molecule cytokines, such as IL-17 and TGF-β, play a role in immune regulation, small-molecule metabolites with molecular weights ranging from 50 to 1500 Da have the capacity to more readily penetrate cell membranes and modulate *CD4*^*+*^ *T* cell differentiation pathways. The quality control data is shown in Figure S5. As illustrated in Fig. 8A, treatment with CSp-OxD@Cu^2+^/G resulted in significant alterations in the principal components of metabolites in LPS-treated *RAW264.7* cells. By applying criteria of a fold change greater than 2 and a p-value less than 0.05, we identified 10 metabolites that are highly associated with inflammatory and redox processes among the upregulated metabolites, including betamethasone acetate, uric acid, and the reduced form of glutathione (Fig. 8B). Of these metabolites, only uric acid has been clearly evidenced as being secreted by *RAW264.7* cells, with potential implications for the *CD4*^*+*^ *T* cell environment. Furthermore, Figs. 8C, D present the cluster heatmap at the class level and the KEGG enrichment analysis of the differentially expressed metabolites, respectively.

We measured intracellular and extracellular uric acid concentrations of *RAW264.7* cells under different treatments, with the observed trends consistent with non-targeted metabolomics results (Fig. 8E). Subsequently, we examined the impact of uric acid at physiological concentrations on *CD4*^*+*^ *T* cells. As illustrated in Fig. 8F, uric acid at these concentrations modulates the differentiation of *CD4*^*+*^ *T* cells towards an anti-inflammatory phenotype. This modulation is evidenced by the suppression of pro-inflammatory cytokines (IL-17, IL-2, IL-21) and the enhancement of anti-inflammatory cytokines (IL-4, IL-10, TGF-β), suggesting that uric acid may play a crucial role in the cross-talk between macrophages and *CD4*^*+*^ *T* cells.

### CSp-OxD@Cu^2+^/G hydrogel enhances wound healing in diabetic mice

**Fig. 9:**
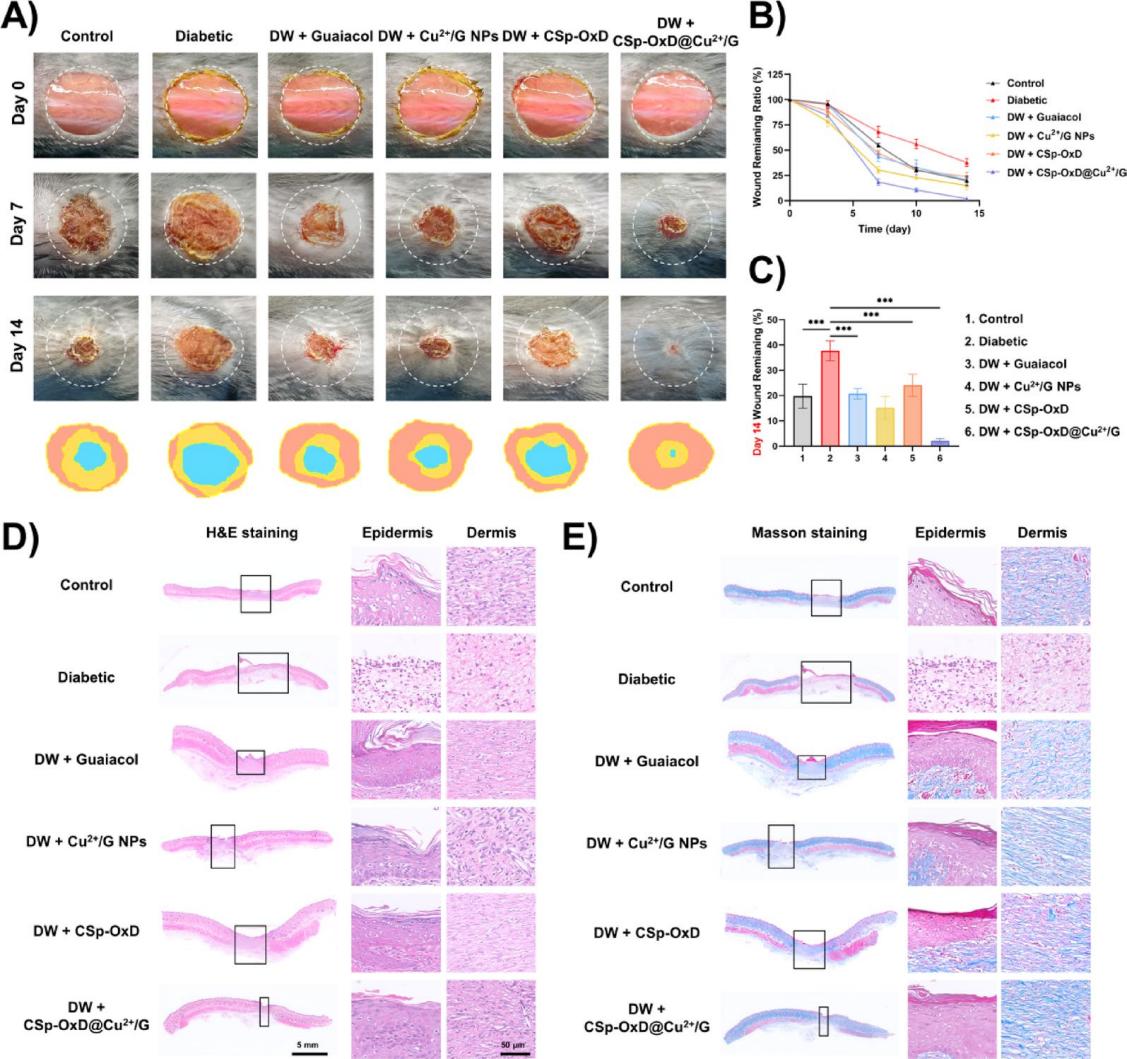
CSp-OxD@Cu^2+^/G hydrogel enhances wound healing in diabetic mice. **A**) Wound images at days 0, 7, and 14 for different treatments; **B**) Healing rates at days 3, 7, 10, and 14; **C**) remaining wound rate, **D**) H&E staining images, and **E**) Masson staining images under different treatments at day 14. Data are presented as mean ± SD, *n* = 6; *P < 0.05, **P < 0.01, ***P < 0.001, and ns, not significant

To evaluate the efficacy of CSp-OxD@Cu^2+^/G hydrogel in enhancing wound healing in diabetic wounds in vitro, we induced wounds on the dorsal surface of diabetic mice and administered various treatments. This hydrogel system degrades rapidly under weakly acidic conditions, with approximately 80% degradation occurring within 5 days, during which sustained drug release takes place. Accordingly, drug administration was performed on days 0, 3, 7, and 10. Figure 9A presents representative images of the wounds at baseline (day 0), and at days 7 and 14 post-treatment. The application of CSp-OxD@Cu^2+^/G hydrogel markedly accelerated wound healing in diabetic wounds, as indicated by a significantly reduced wound remaining rate compared to other experimental groups, particularly noticeable by day 14 (Fig. 9B, C). To further investigate, tissue staining was employed to evaluate epithelial regeneration and collagen deposition in diabetic wounds on day 14. H&E staining revealed that treatment with CSp-OxD@Cu^2+^/G hydrogel significantly enhanced epithelial regeneration, with epithelial thickness substantially greater than that observed in other groups, including the Control group (Fig. 9D, S6A). Similarly, Masson’s trichrome staining demonstrated a pronounced increase in collagen deposition in the CSp-OxD@Cu^2+^/G hydrogel group (Fig. 9E, S6B).

### CSp-OxD@Cu^2+^/G hydrogel harmonizes macrophage and Th17/Treg balancing in diabetic wound healing

**Fig. 10:**
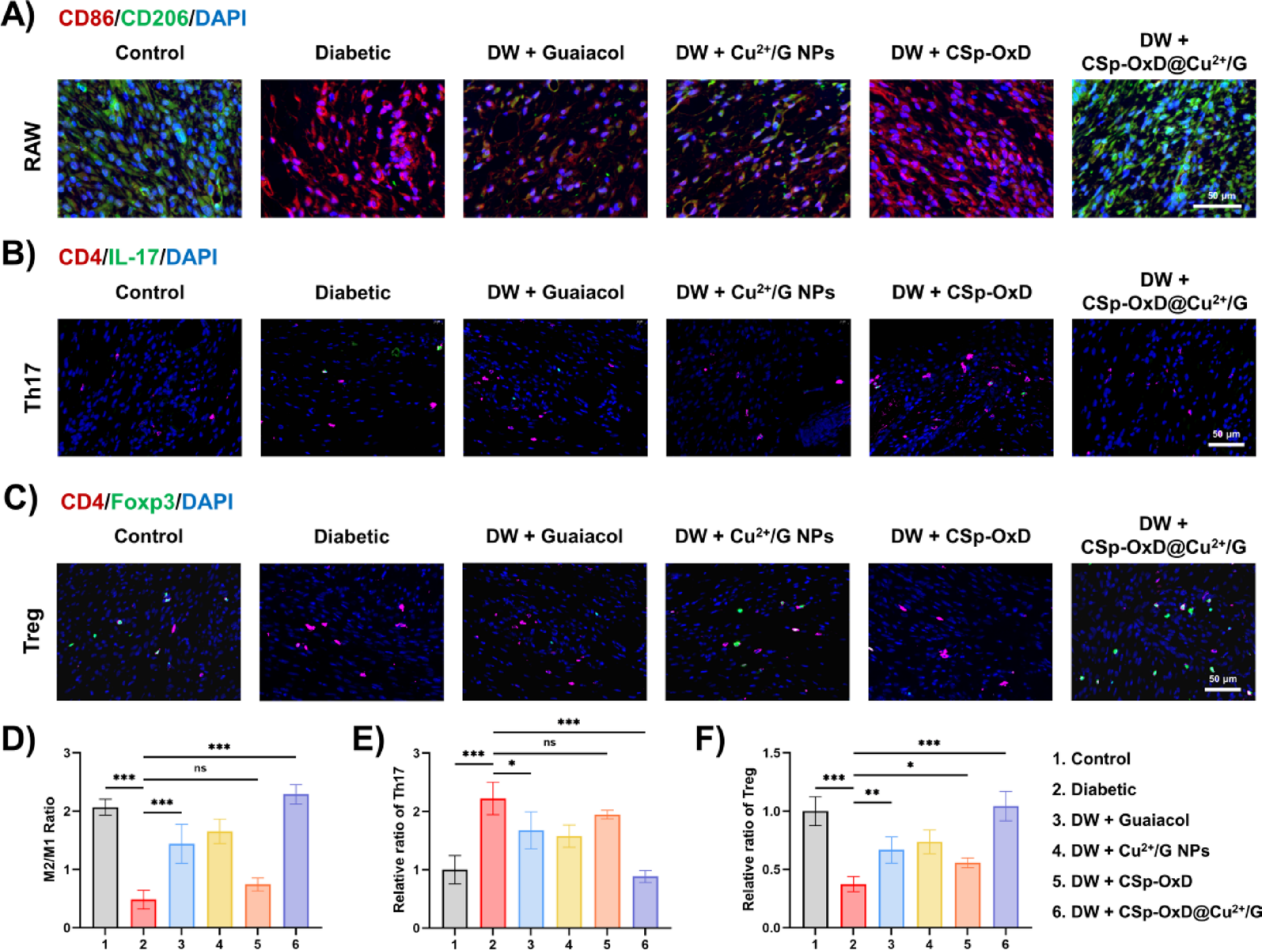
The CSp-OxD@Cu^2+^/G hydrogel modulates the immune environment in diabetic mice wounds. **A**) Fluorescence images and **D**) analysis of CD86/CD206/DAPI staining in mouse wound tissue on day 7 with various treatments; **B**) Fluorescence images and **E**) analysis of CD4/IL-17/DAPI staining in mouse wound tissue on day 7 with various treatments; **C**) Fluorescence images and **F**) analysis of CD4/Foxp3/DAPI staining in mouse wound tissue on day 7 with various treatments. Data are presented as mean ± SD, *n* = 6; *P < 0.05, **P < 0.01, ***P < 0.001, and ns, not significant

Following the demonstration that the CSp-OxD@Cu^2+^/G hydrogel facilitates wound healing in diabetic wounds, we conducted further investigations into its effects on modulating the immune microenvironment. To assess immune infiltration in the wound tissues of diabetic mice on day 7, immunofluorescence staining was employed. We focused on macrophages and Th17/Treg cells, which are critical components of adaptive and specific immunity, respectively, to evaluate the overall immune status of the wound tissue. CD86 and CD206 markers were utilized to identify M1 and M2 macrophages, respectively. As illustrated in Fig. 10A, D, the M2/M1 macrophage ratio in diabetic wounds treated with the CSp-OxD@Cu^2+^/G hydrogel (group 6) was significantly elevated compared to the untreated group (group 2) and was nearly equivalent to that of the control group (group 1). These findings suggest that the CSp-OxD@Cu^2+^/G hydrogel is capable of ameliorating the disruption of local adaptive immunity in diabetic wounds, effectively restoring it to normal levels. In *CD4*^*+*^ *T* cells, the Th17 and Treg cell subtypes are pivotal in directing the progression of inflammatory responses. Th17 cells are identified by the CD4/IL-17 marker, while Treg cells are indicated by the CD4/Foxp3 marker. As illustrated in Figs. 10B, E, the application of the CSp-OxD@Cu^2+^/G hydrogel (group 6) resulted in a significant reduction in the relative proportion of Th17 cells within diabetic wounds. This finding implies that the hydrogel system facilitates the anti-inflammatory progression in diabetic wounds. Similarly, the proportion of Treg cells exhibited a notable increase, with levels in diabetic wounds significantly rising post-hydrogel treatment (group 6) and returning to those observed in the control group (group 1) (Fig. 10C, F). The original image of immunofluorescence staining is shown in Figure S7-S9. qRT-PCR results were consistent with immunofluorescence staining findings. Treatment with CSp-OxD@Cu^2+^/G NPs downregulated the expression of IL-17 and RORγt in diabetic wound tissues, indicating a reduction in Th17 cells, while upregulating the expression of Foxp3 and CD25, suggesting an increase in Treg cells (Figure S16).

Furthermore, we investigated pyroptosis-related gene expression and uric acid levels in the wound tissues. qRT-PCR analysis revealed that CSp-OxD@Cu^2+^/G treatment significantly decreased the expression of NLRP3 and Caspase-1 in diabetic wounds, demonstrating an overall attenuation of pyroptosis in the skin tissue (Figure S17). Although no significant differences were observed in the qRT-PCR results for IL-1β and GSDMD, their changes, as suggested by prior in vitro experiments, might occur at the level of downstream protein cleavage, warranting further analysis by Western blot. Concurrently, CSp-OxD@Cu^2+^/G treatment elevated uric acid levels in the wounds, corroborating the cellular experimental results from this study (Figure S18). It should be noted that both qRT-PCR and uric acid measurements reflect the overall tissue status. Precise in vivo assessment of pyroptosis, cell population counts, and metabolic activity within specific cell types requires further investigation.

In conclusion, the CSp-OxD@Cu^2+^/G hydrogel effectively modulates macrophage infiltration and the Th17/Treg balance in diabetic wounds on the dorsal wound of mice.

## Conclusion

The CSp-OxD@Cu^2+^/G smart hydrogel system developed in this study effectively modulates the immune microenvironment of diabetic wounds by incorporating pH-responsive and dual-dynamically crosslinked hydrogels with Cu²⁺/G nanoparticles. This system facilitates the polarization of macrophages towards the M2 anti-inflammatory phenotype through the sustained release of guaiacol and Cu²⁺, thereby inhibiting pyroptosis and restoring energy metabolic homeostasis. Furthermore, it indirectly regulates the Th17/Treg balance of *CD4+ T* cells via macrophage interaction, thereby simultaneously remodeling both innate and adaptive immune networks. Metabolomic analyses indicate that uric acid may play a role in macrophage-*CD4+ T* cell communication. In vivo experiments demonstrate that the CSp-OxD@Cu²⁺/G hydrogel significantly accelerates wound healing in diabetic mice, enhances epithelial regeneration and collagen deposition, and effectively restores local immune homeostasis. Notably, the relatively rapid degradation rate of this hydrogel system necessitates multiple administrations in practical applications, which may present challenges. Achieving a balance between environmental responsiveness and extended degradation duration, optimally aligned with the treatment cycle of diabetic wounds, will be a key focus of our subsequent research. This study not only offers a novel perspective for understanding the mechanisms underlying immune imbalance in diabetic wounds but also establishes a theoretical and experimental foundation for the development of multifunctional wound dressings that target the immune microenvironment, demonstrating broad clinical translation potential.

## Supplementary Information

Supplementary Material 1

## Data Availability

No datasets were generated or analysed during the current study.
