# Supplementary material for: pH-responsive and dual-dynamically crosslinked metal-phenolic hydrogel for synergistic macrophage and Th17/Treg reprogramming in diabetic wounds
Source: J Nanobiotechnology. 2026 Jan 12;24:139. doi: 10.1186/s12951-025-03987-7 (PMC12888242; doi:10.1186/s12951-025-03987-7)
Supplement: Supplementary file 1 — Supplementary Material 1 [file 12951_2025_3987_MOESM1_ESM.docx]

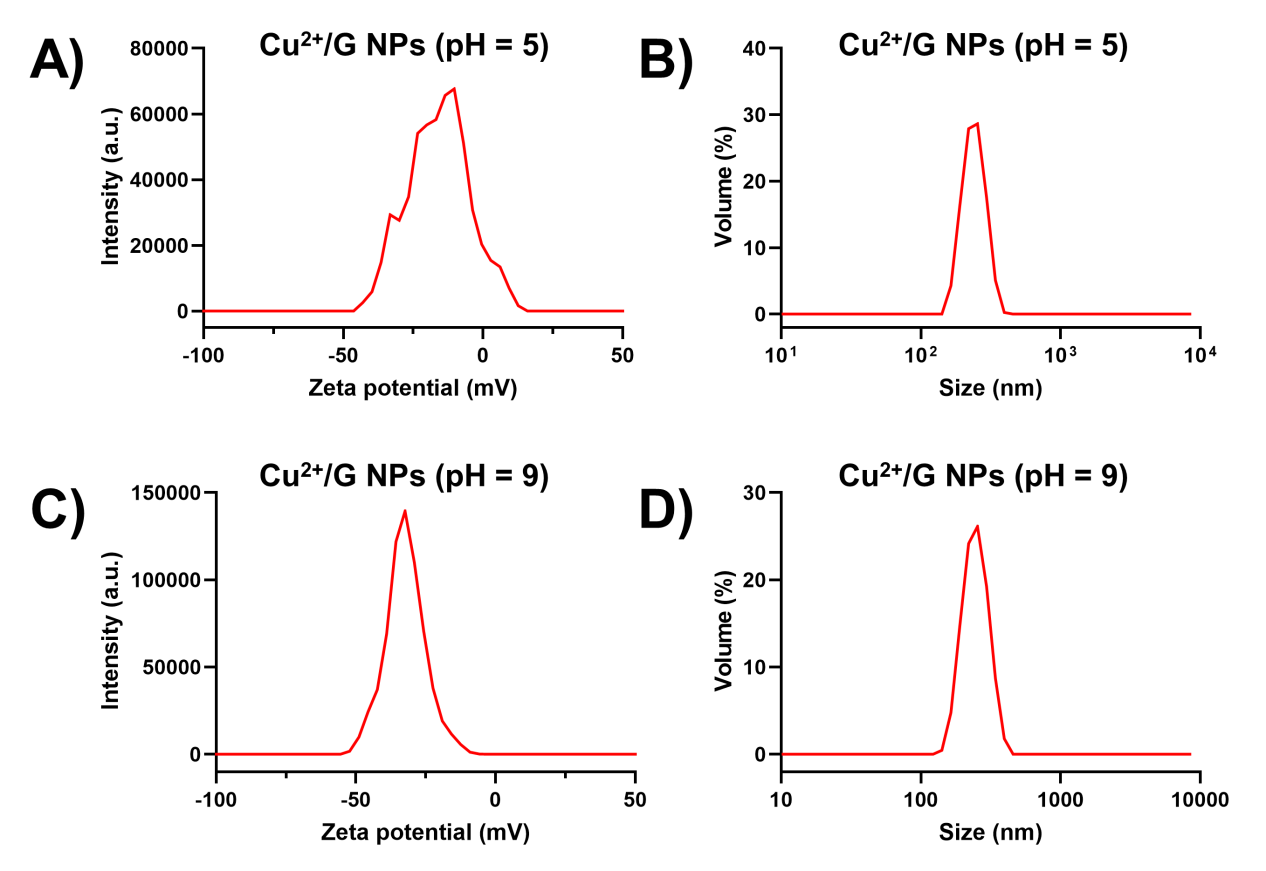


Figure S1. Zeta potential and particle size analysis of Cu^2+^/G NPs in weakly acidic and weakly alkaline environments. A) Zeta potential and B) particle size analysis of Cu^2+^/G NPs at pH = 5; C) Zeta potential and D) particle size analysis of Cu^2+^/G NPs at pH = 9.


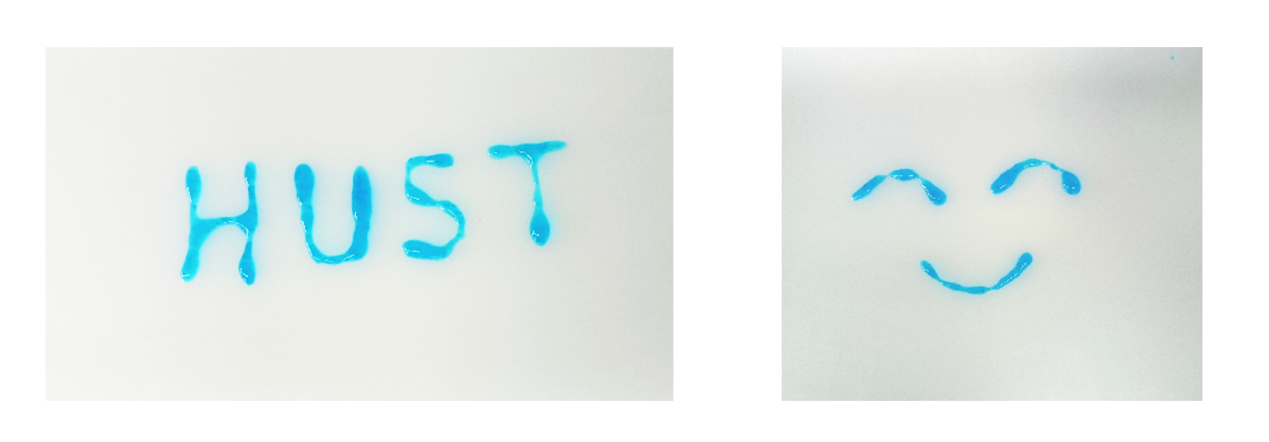


Figure S2. CSp-OxD hydrogel was injected into corresponding patterns.


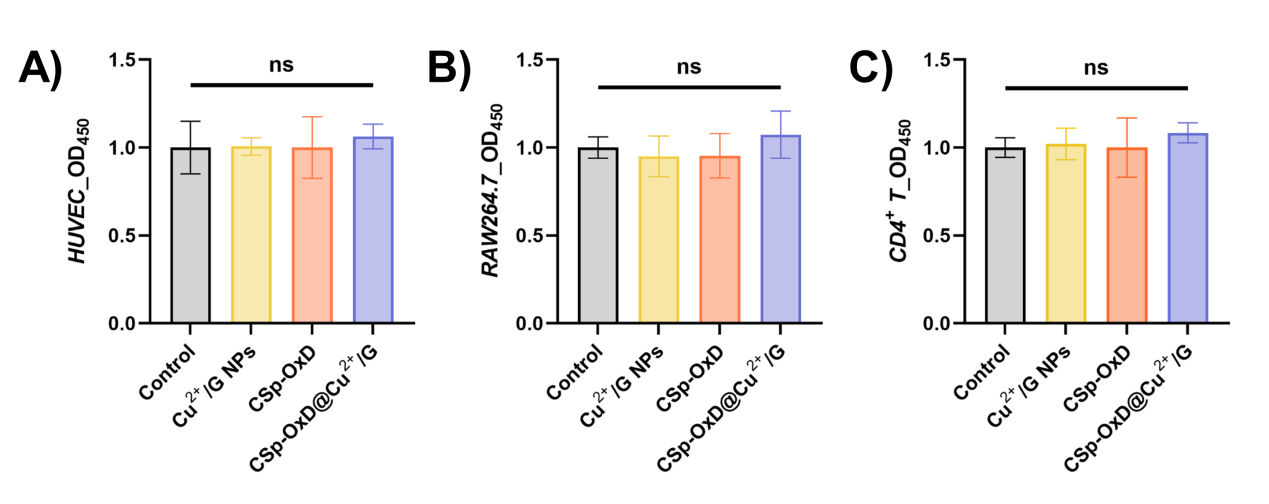


Figure S3. Absorbance at 450 nm for CCK8 experiments with A) *HUVEC*s, B) *RAW264.7* and C) *CD4^+^ T* cells under different treatments. (*P < 0.05, **P < 0.01, ***P < 0.001 and ns, not significant)


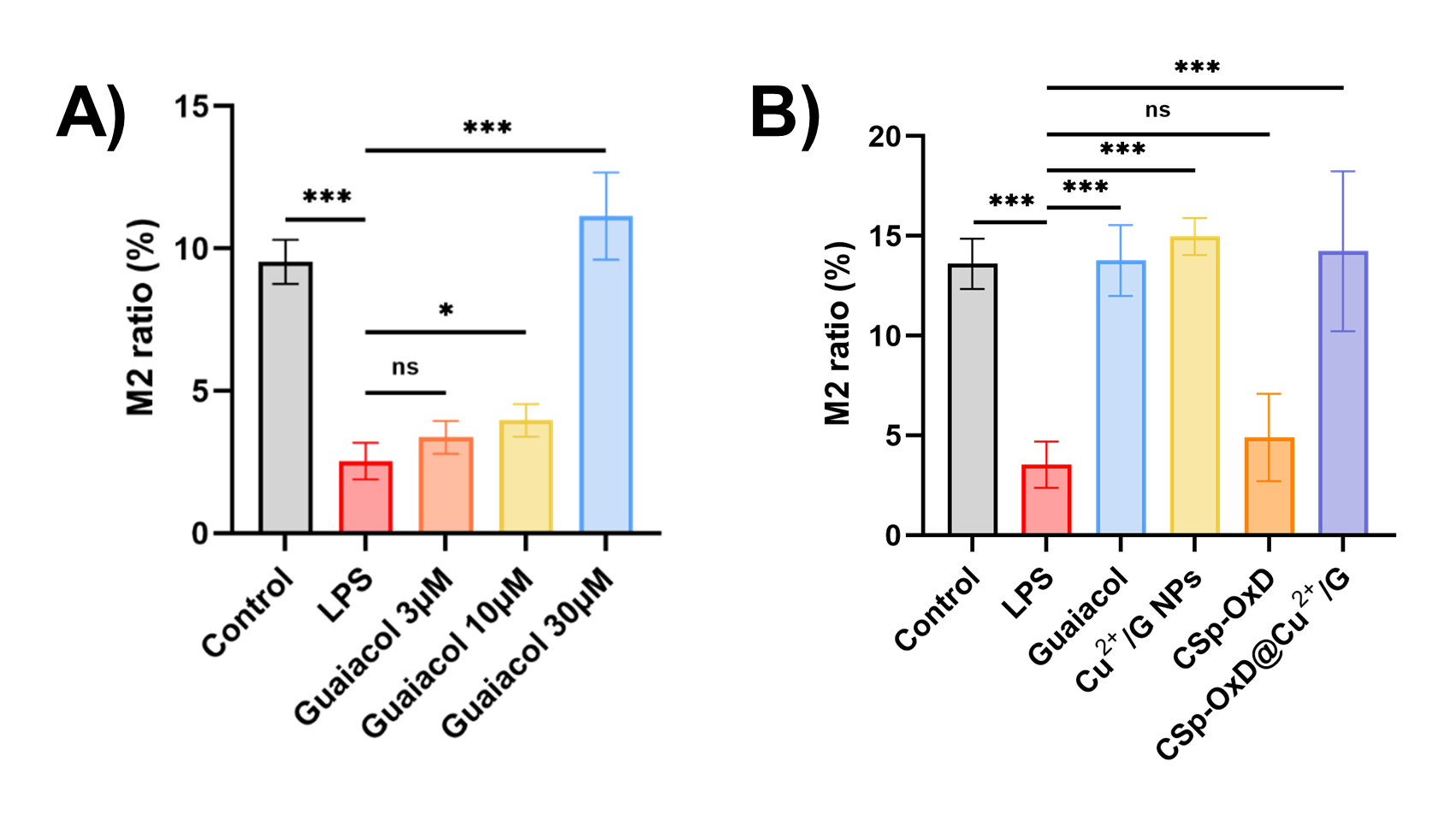


Figure S4. A) Flow cytometry analysis of *RAW264.7* treated with guaiacol at different concentrations; B) Flow cytometry analysis of *RAW264.7* after different treatments. (*P < 0.05, **P < 0.01, ***P < 0.001 and ns, not significant)


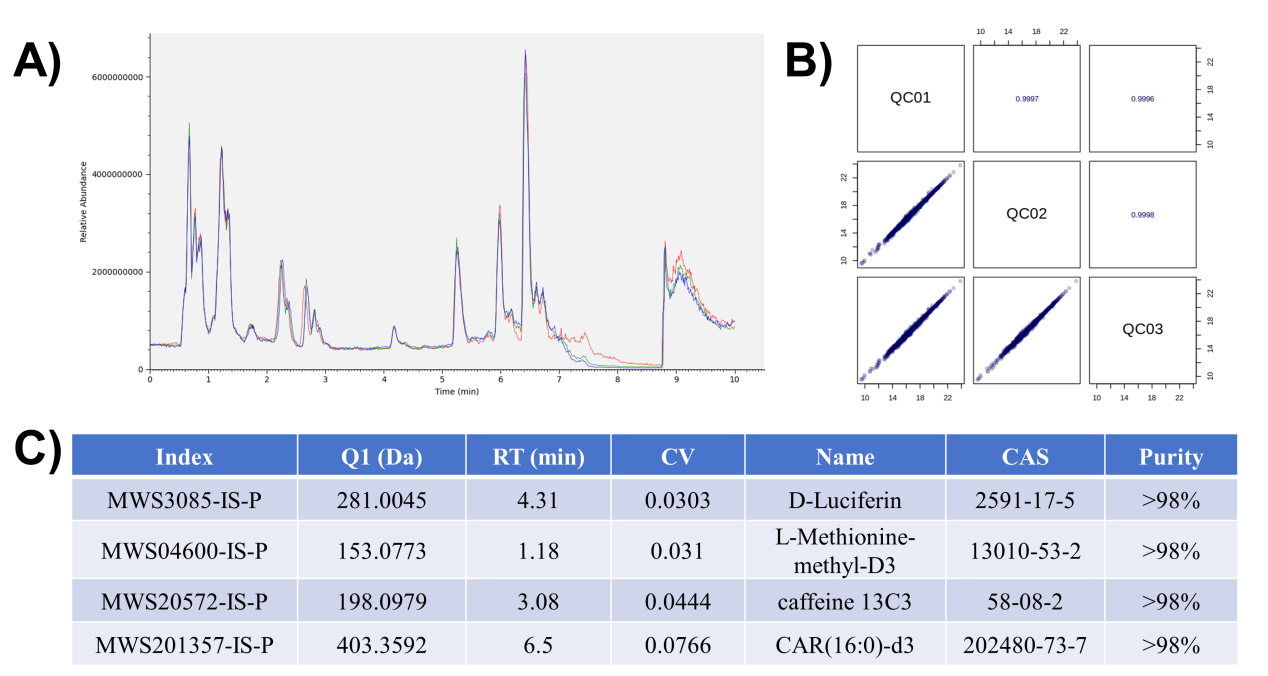


Figure S5. Quality control analysis of non-targeted metabolomics. A) Overlay plot of total ion chromatogram (TIC) for QC sample mass spectrometry detection; B) Pearson correlation analysis of QC samples; C) Stability of internal standards in QC samples.


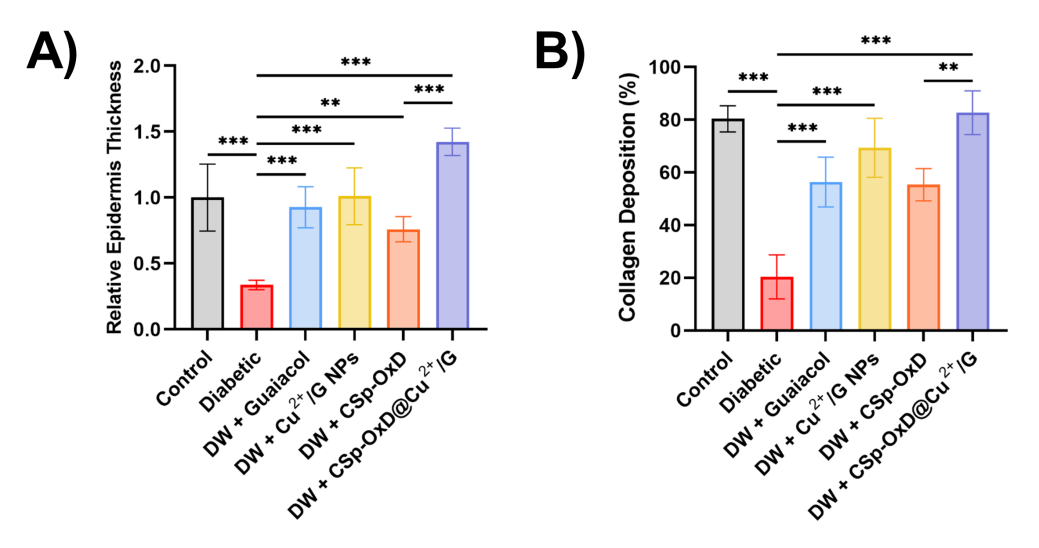


Figure S6. CSp-OxD@Cu^2+^/G hydrogel enhances wound healing in diabetic mice. Statistical analysis of A) epithelialization and B) collagen deposition in wounds of mice in different treatment groups. (*P < 0.05, **P < 0.01, ***P < 0.001 and ns, not significant)


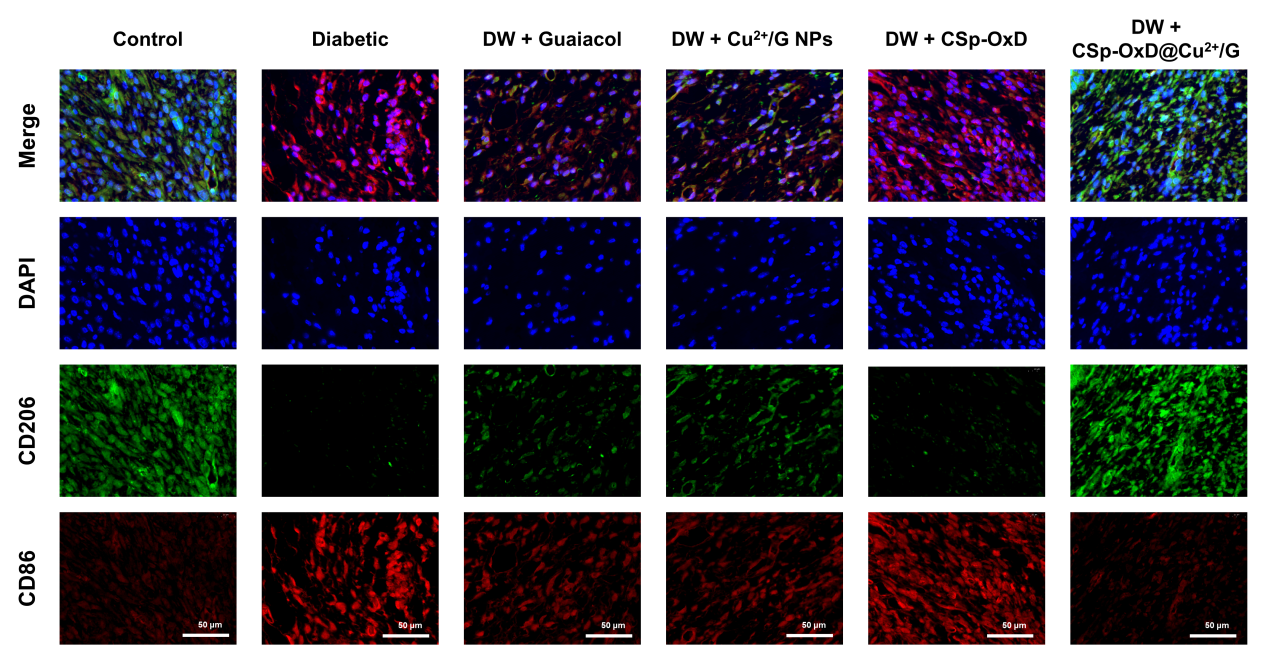


Figure S7. Original image of immunofluorescence staining (DAPI/CD86/CD206) characterizing the status of macrophages.


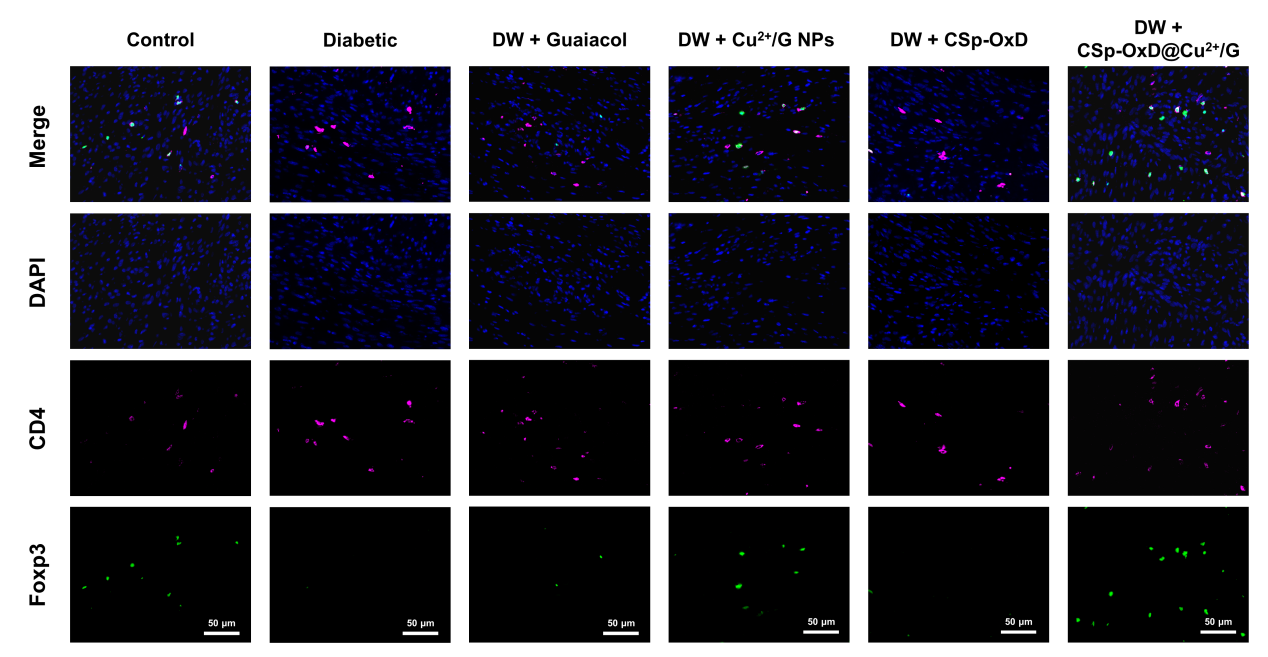


Figure S8. Original image of immunofluorescence staining (DAPI/CD4/Foxp3) characterizing the status of wound Treg cells.


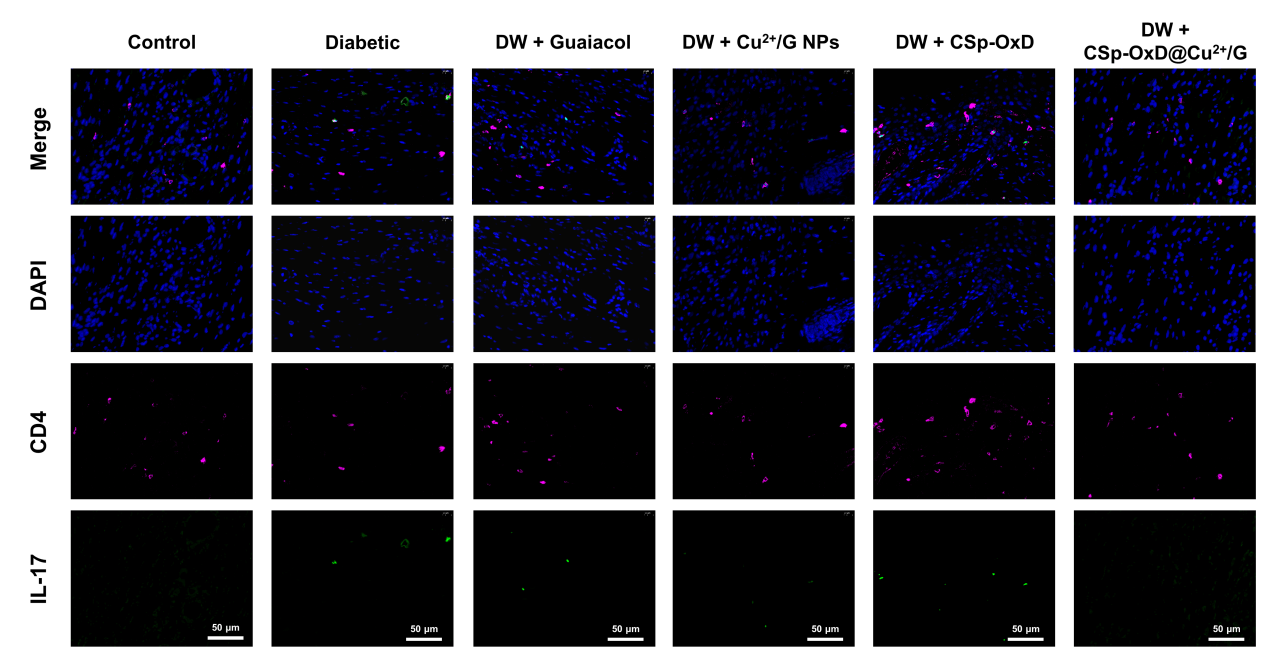


Figure S9. Original image of immunofluorescence staining (DAPI/CD4/IL-17) characterizing the status of wound Th17 cells.


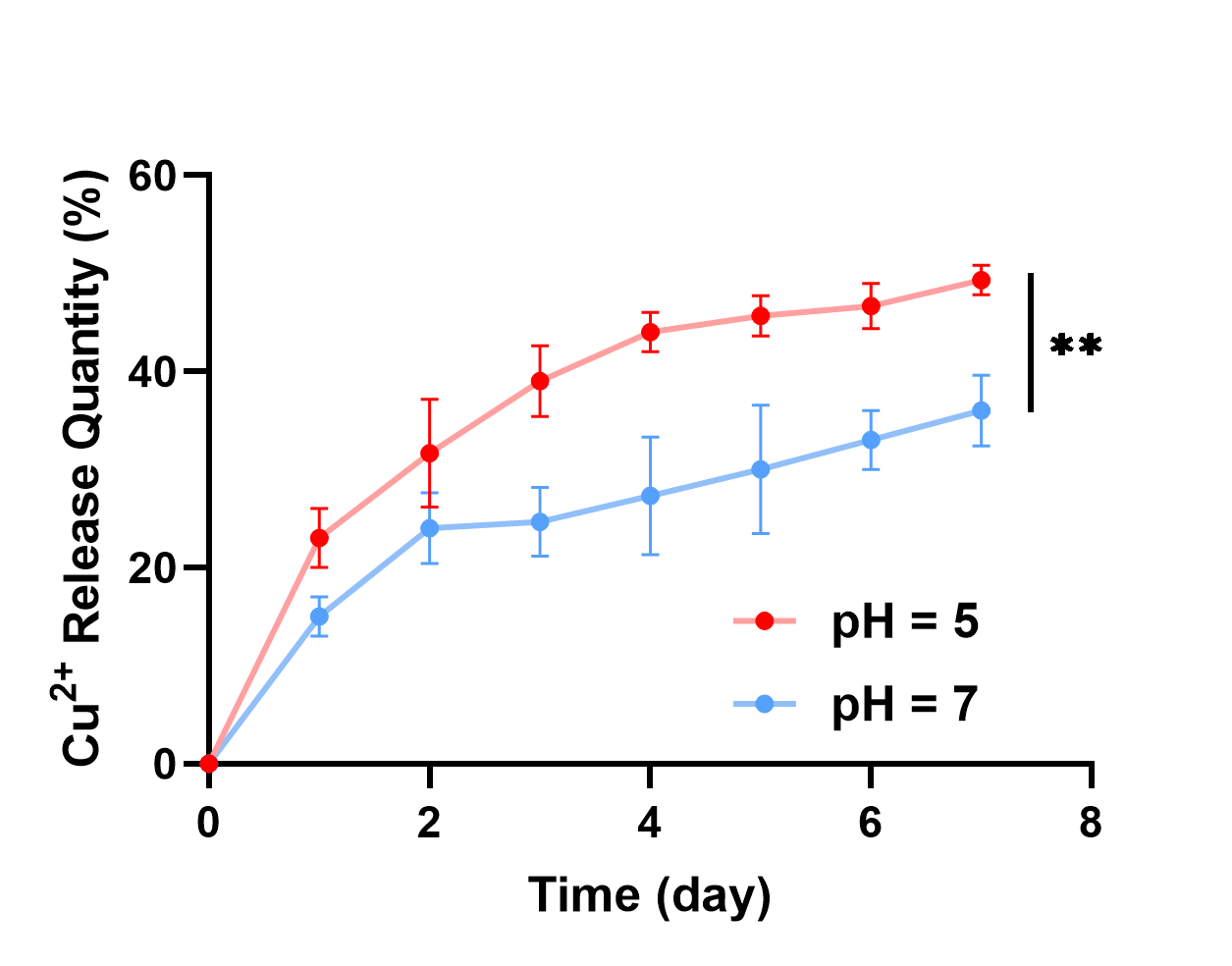


Figure S10. Time dependent curves of Cu^2+^ release from CSp-OxD@Cu^2+^/G hydrogel in buffer solutions of different pH values.


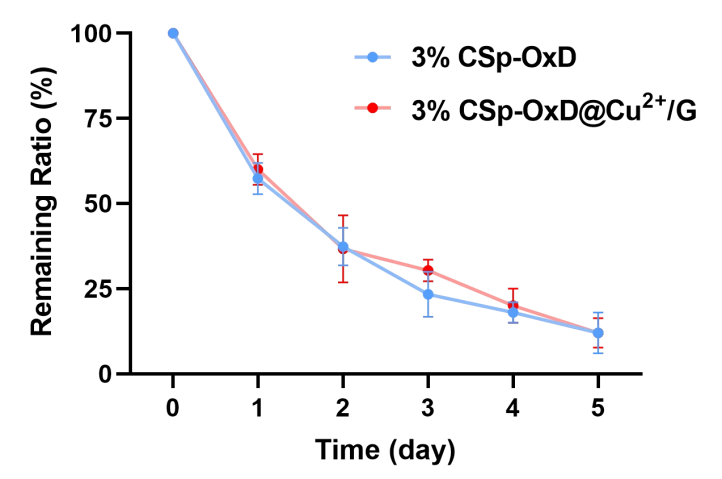


Figure S11. Degradation characteristics of CSp-OxD and CSp-OxD@Cu^2+^/G hydrogels in vivo.


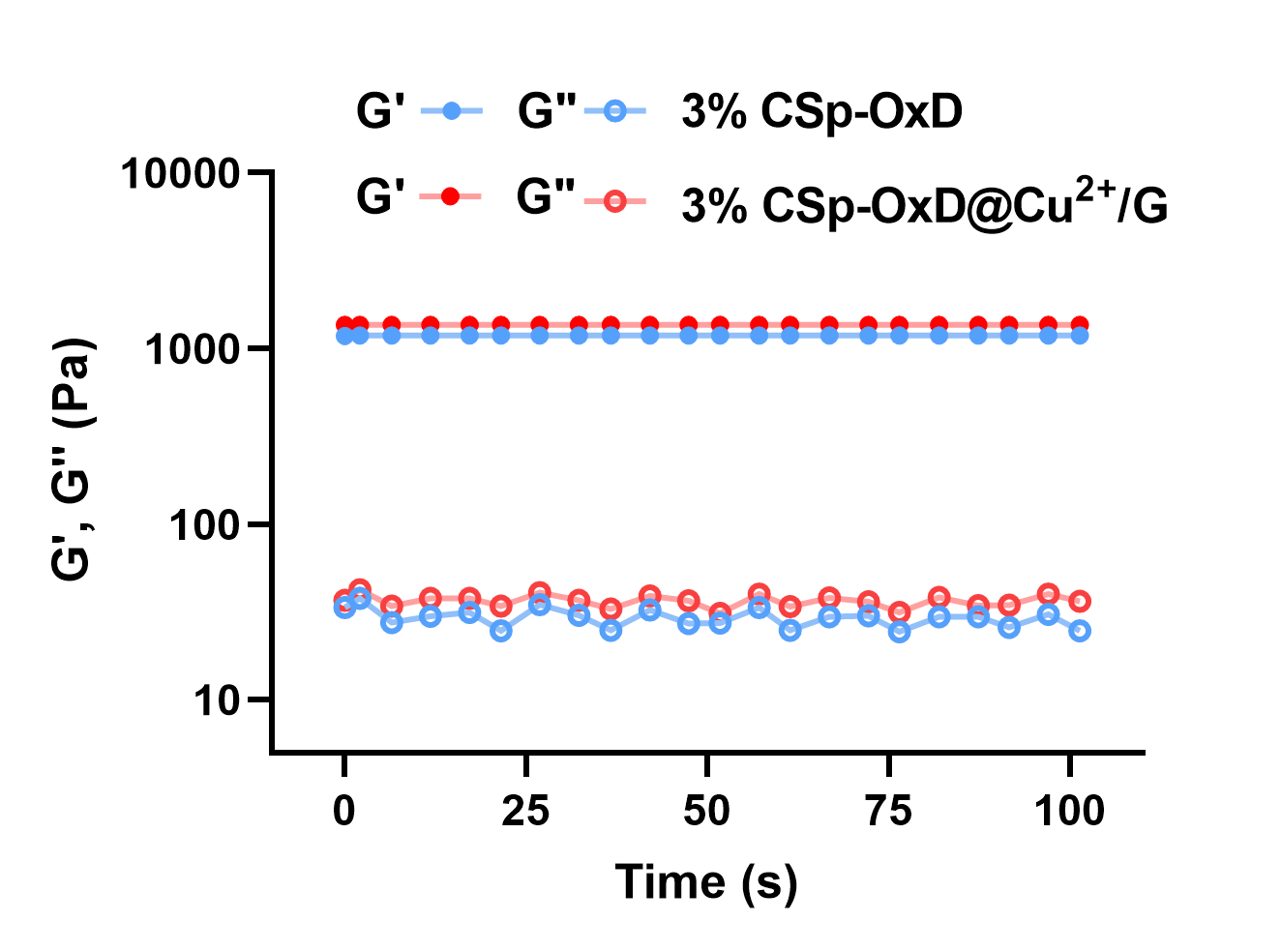


Figure S12. Rheological test results of CSp-OxD and CSp-OxD@Cu^2+^/G hydrogels.


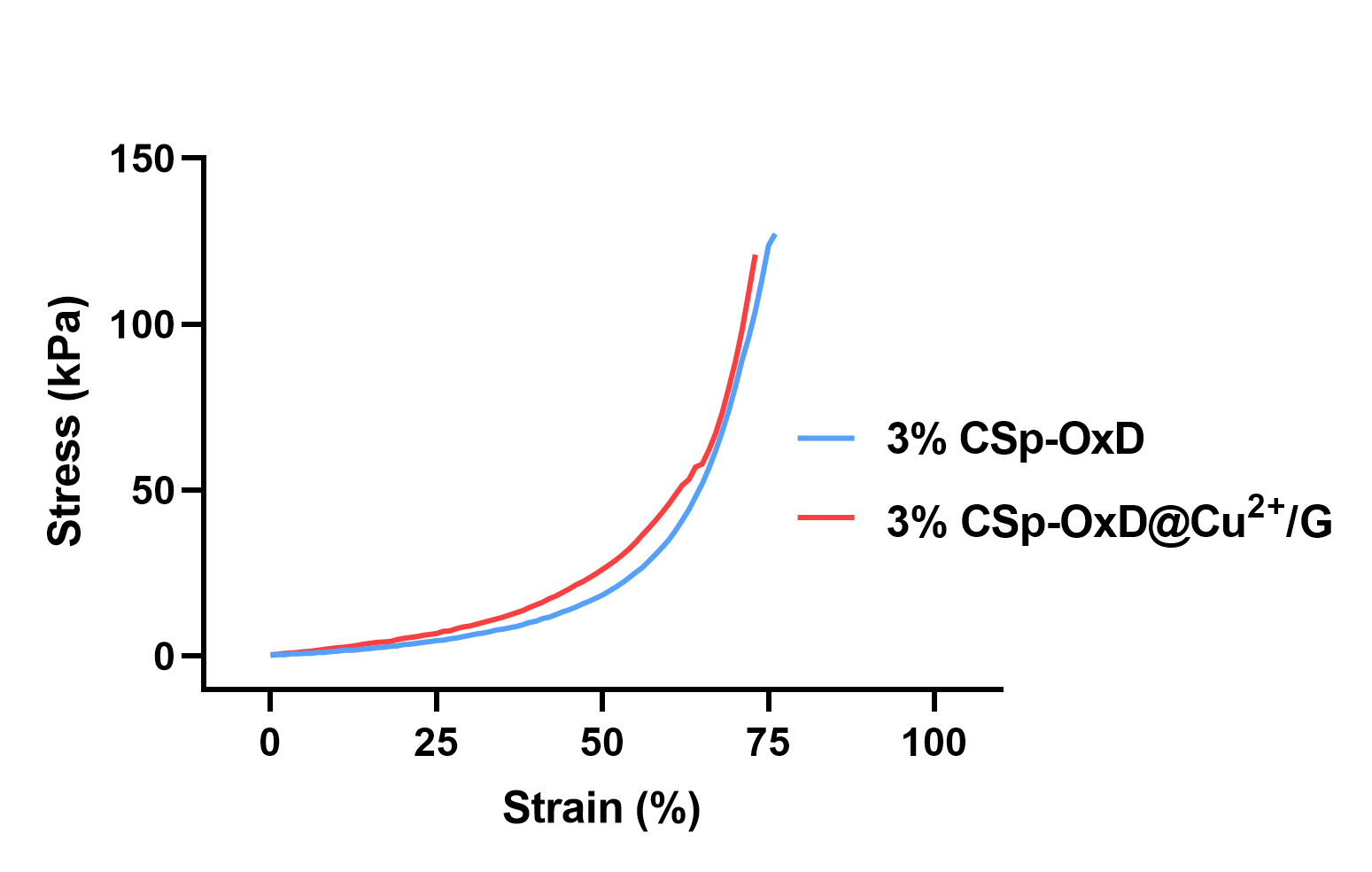


Figure S13. Compression test results of CSp-OxD and CSp-OxD@Cu^2+^/G hydrogels.


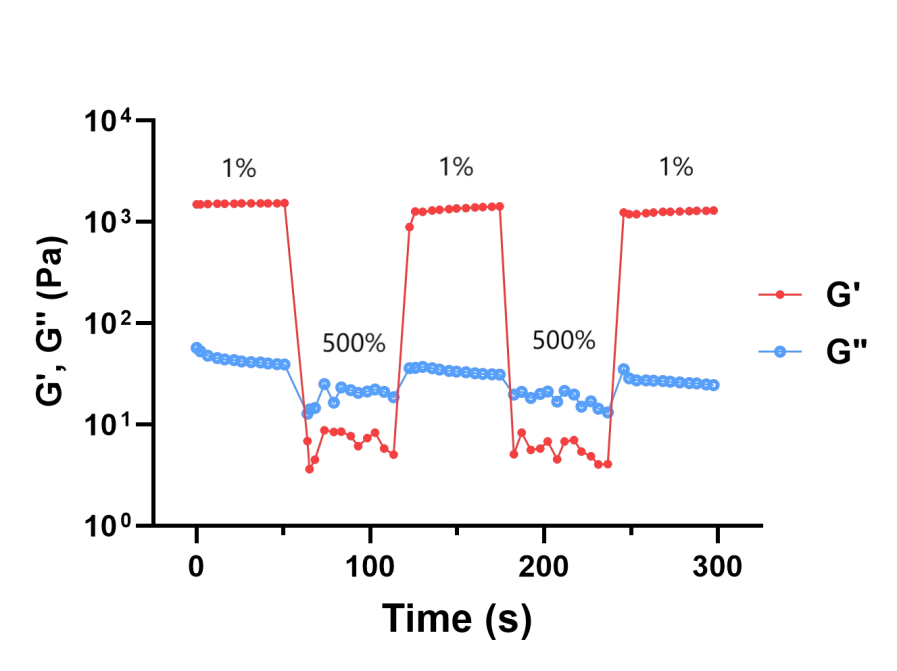


Figure S14. Step-strain test results of 3% CSp-OxD@Cu^2+^/G hydrogel.


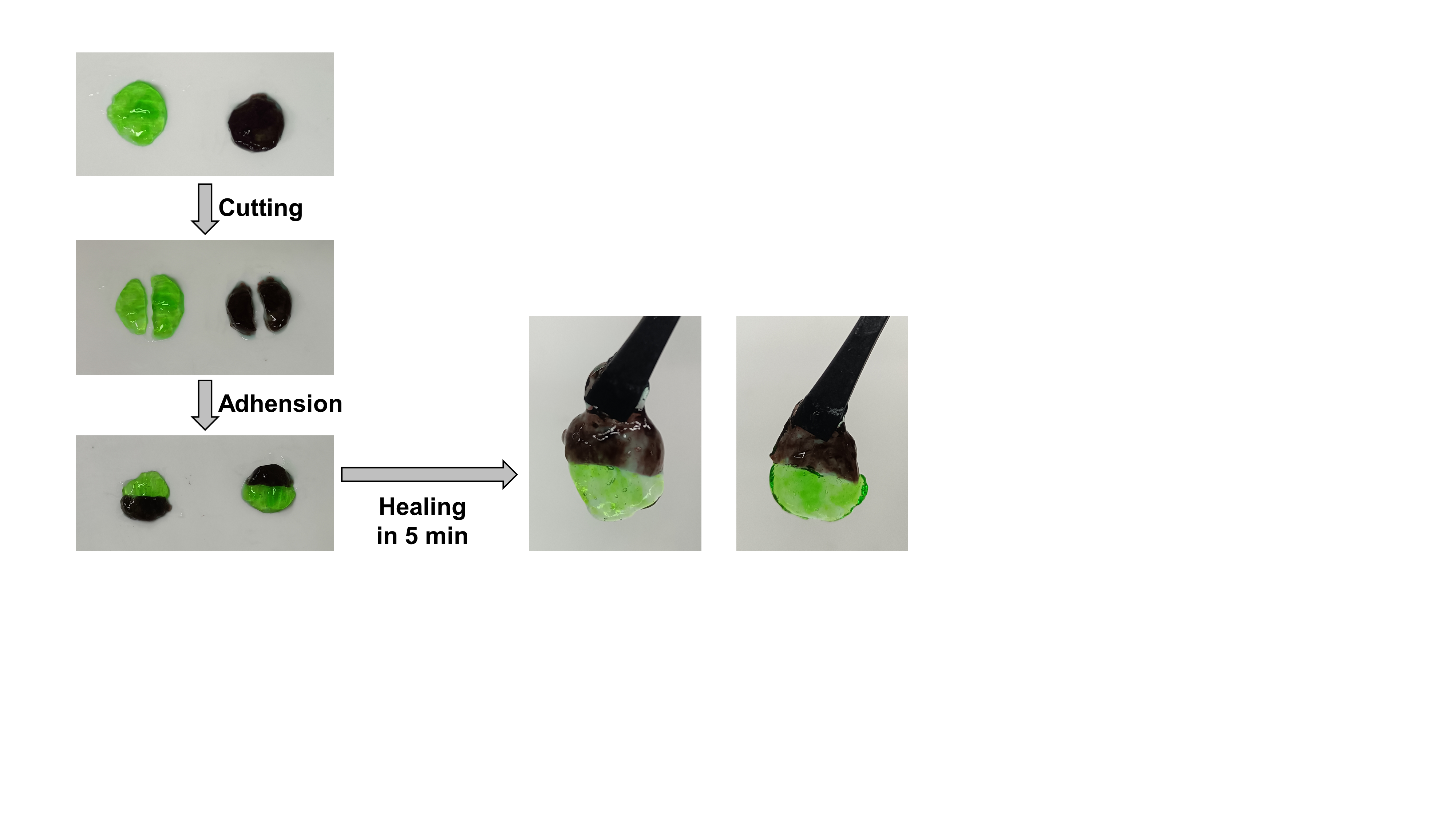


Figure S15. Self-healing behaviour of CSp-OxD hydrogel.


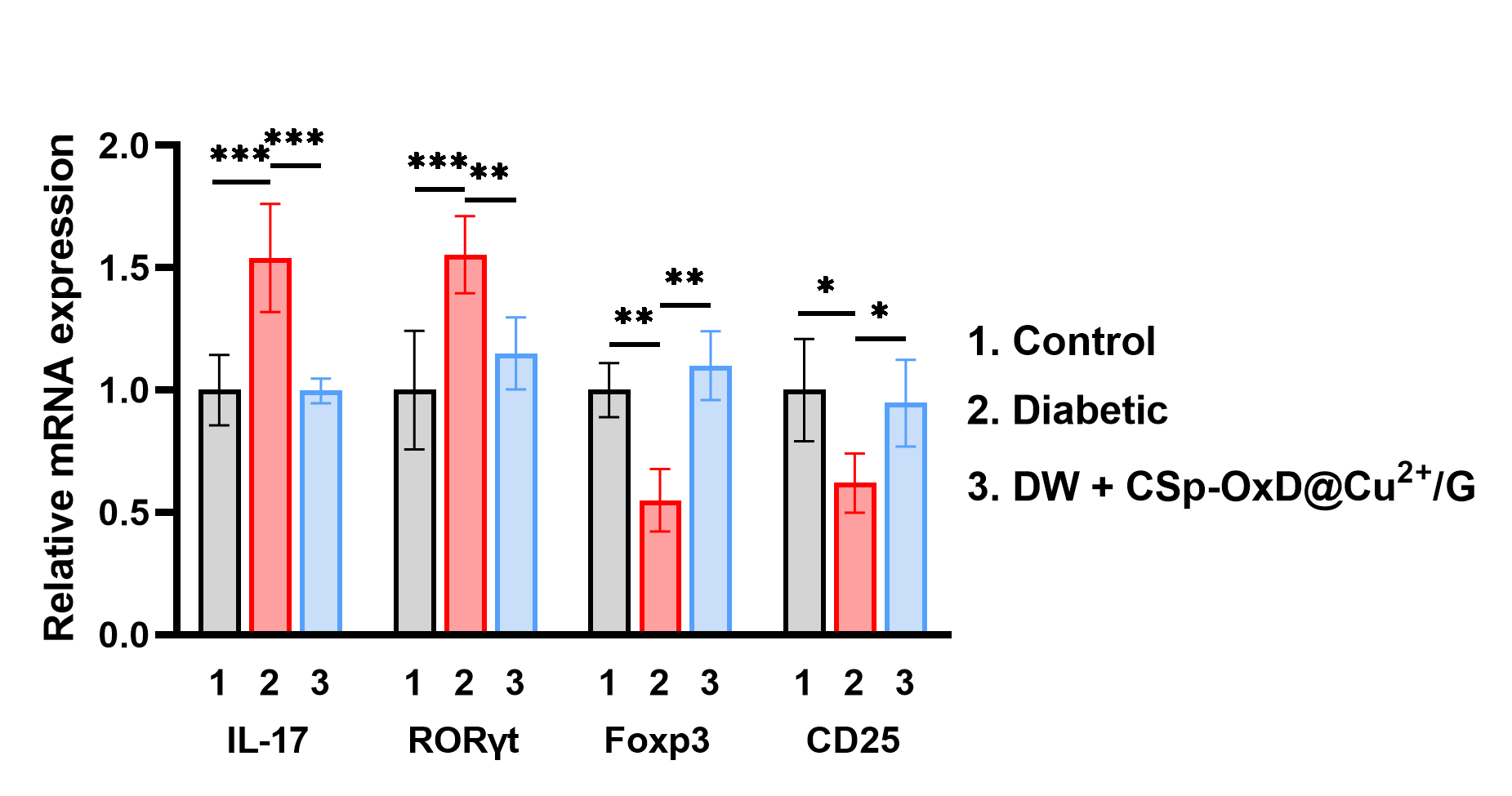


Figure S16. qRT-PCR analysis of Th17- and Treg-related gene expression in wound tissues from different treatment groups.


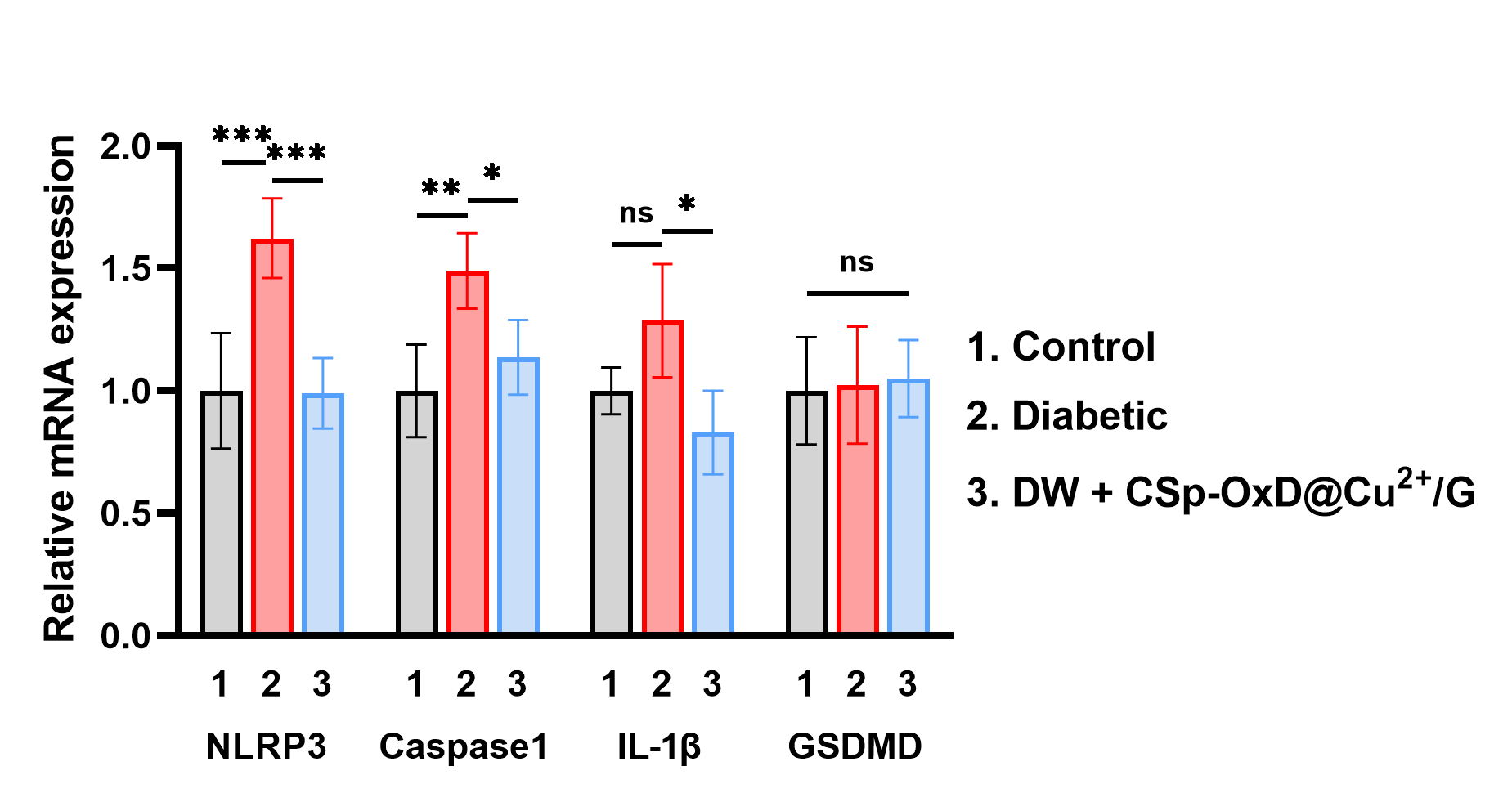


Figure S17. qRT-PCR analysis of pyroptosis-related gene expression in wound tissues from different treatment groups.


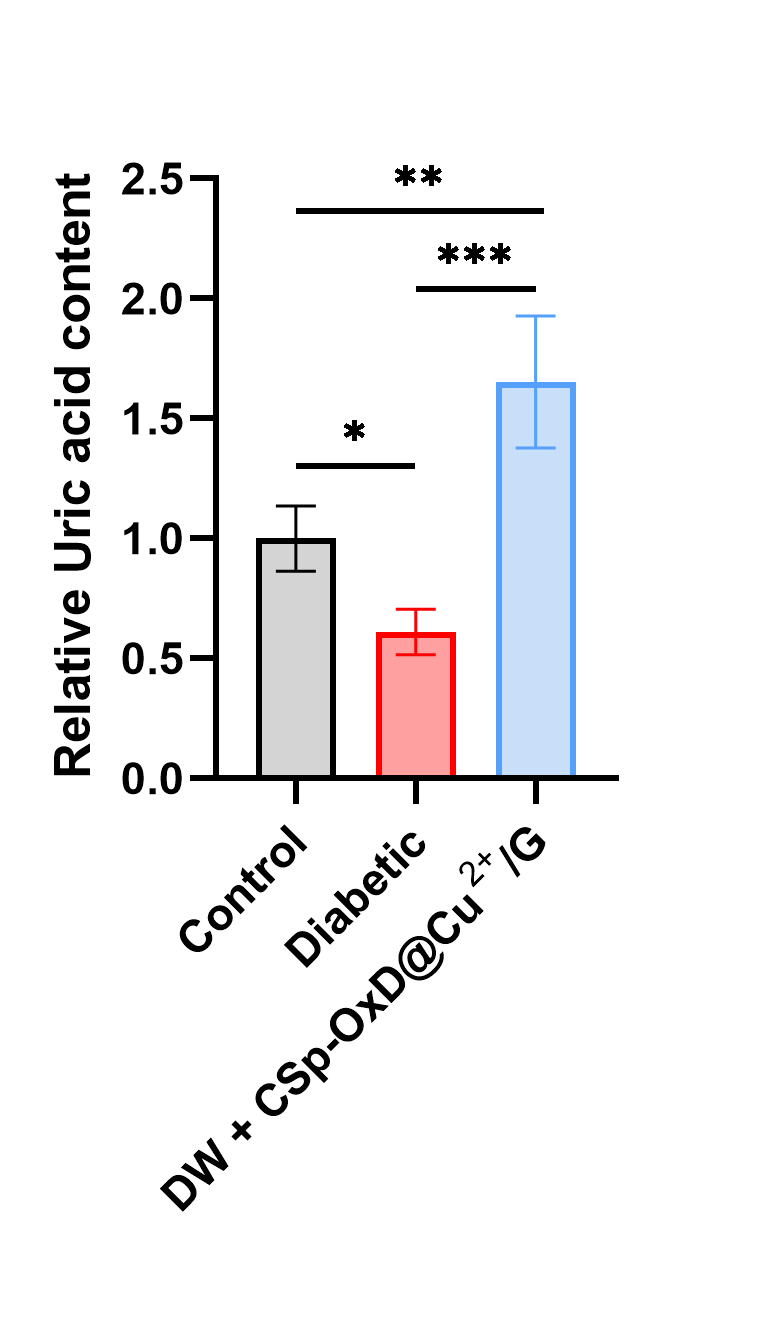


Figure S18. Uric acid levels in wound tissues from different treatment groups.


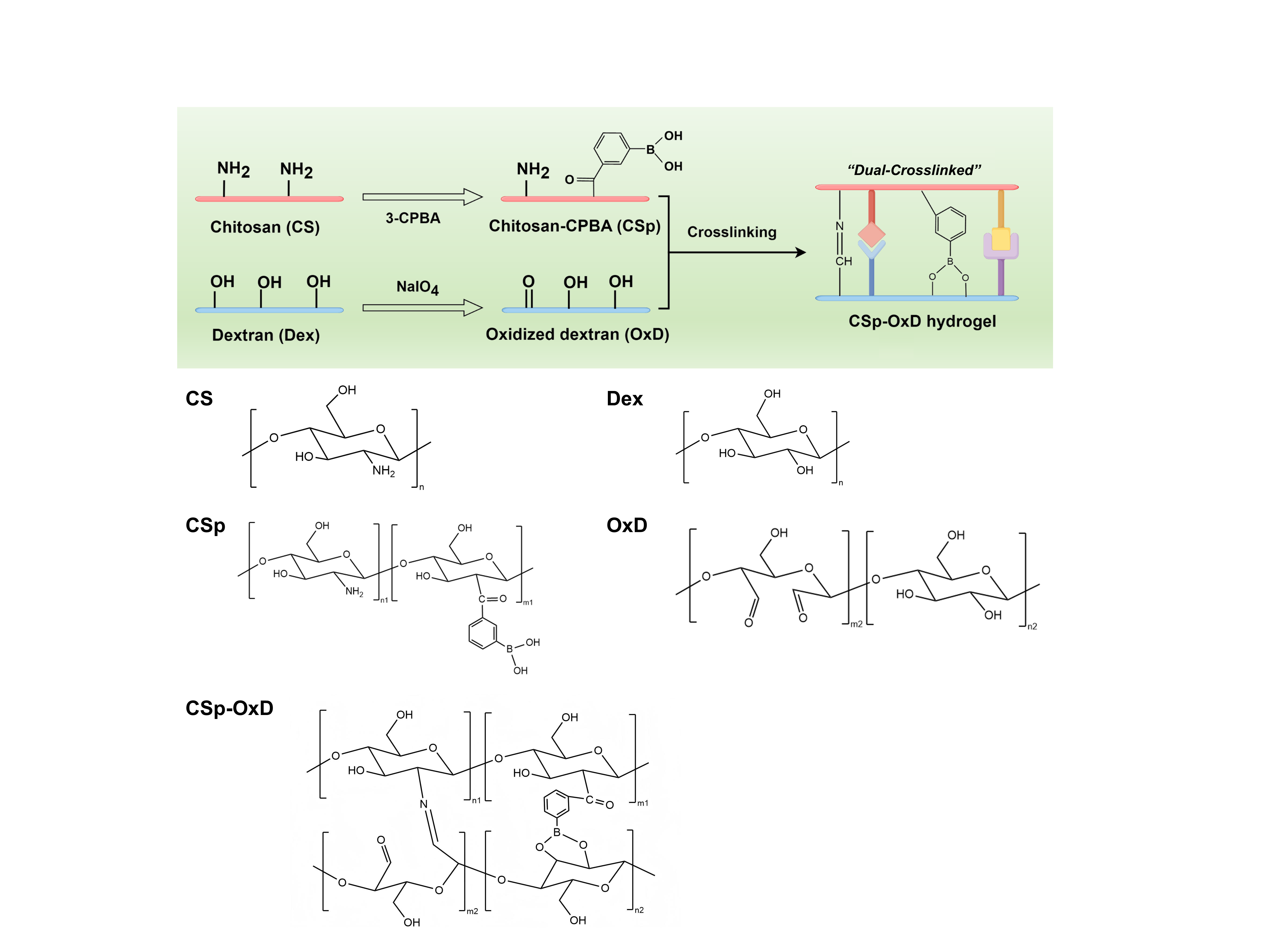


Figure S19. Chemical structure corresponding to hydrogel in Scheme 1.


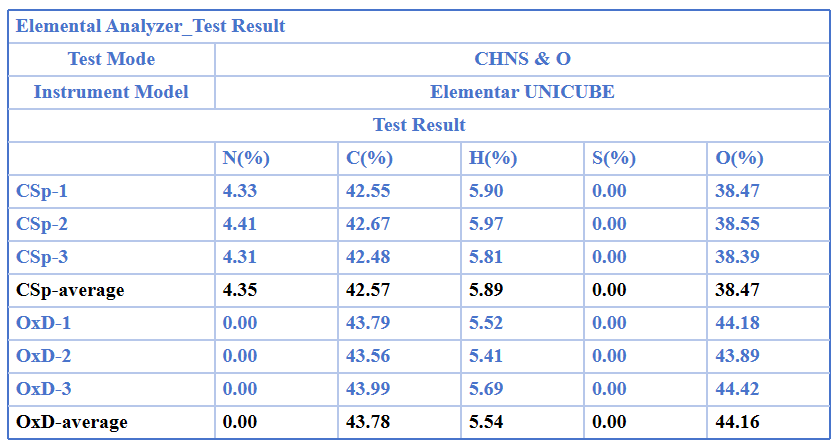


Table S1. The elemental analyzer detection results of CSp and OxD.
